# HyphaTracker: An ImageJ toolbox for time-resolved analysis of spore germination in filamentous fungi

**DOI:** 10.1038/s41598-017-19103-1

**Published:** 2018-01-12

**Authors:** Michael Brunk, Sebastian Sputh, Sören Doose, Sebastian van de Linde, Ulrich Terpitz

**Affiliations:** 1 Department of Biotechnology and Biophysics, Julius-Maximilian University Würzburg, Biocenter - Am Hubland, D-97074 Würzburg, Germany; 2 Present Address: Department of Systemphysiology, Leibniz Institute for Neurobiology, Brenneckestraße 6, D-39118 Magdeburg, Germany; 3 Present Address: Department of Physics, University of Strathclyde, 107 Rottenrow East, Glasgow, G4 0NG UK

## Abstract

The dynamics of early fungal development and its interference with physiological signals and environmental factors is yet poorly understood. Especially computational analysis tools for the evaluation of the process of early spore germination and germ tube formation are still lacking. For the time-resolved analysis of conidia germination of the filamentous ascomycete *Fusarium fujikuroi* we developed a straightforward toolbox implemented in ImageJ. It allows for processing of microscopic acquisitions (movies) of conidial germination starting with drift correction and data reduction prior to germling analysis. From the image time series germling related region of interests (ROIs) are extracted, which are analysed for their area, circularity, and timing. ROIs originating from germlings crossing other hyphae or the image boundaries are omitted during analysis. Each conidium/hypha is identified and related to its origin, thus allowing subsequent categorization. The efficiency of HyphaTracker was proofed and the accuracy was tested on simulated germlings at different signal-to-noise ratios. Bright-field microscopic images of conidial germination of rhodopsin-deficient *F. fujikuroi* mutants and their respective control strains were analysed with HyphaTracker. Consistent with our observation in earlier studies the CarO deficient mutant germinated earlier and grew faster than other, CarO expressing strains.

## Introduction

The dispersal of fungi is ensured by dissemination of asexual spores - so called conidia - that are passively transported in the air. Once displaced, the conidium will only germinate, if the fungus faces convenient growth conditions. The germination starts with the formation of a germ-tube invading the substrate. The uptake of nutrients leads to increased extension rates accelerating the hyphal growth. Therefore, early hyphal development can be described by an exponential growth with constant specific rate while later hyphal growth can be described as linear extension^1,2^. The growth rate of fungal hyphae is an important measure for the effect of ambient factors on fungal growth^3–5^. Indeed, an impressive number of ambient factors influences conidia germination, among them light^6,7^.

Light is an environmental trigger frequently used in the regulation of physiological processes in fungi and accordingly filamentous fungi are equipped with many different photoreceptors^8–11^. These light-perceiving proteins are involved in several important decisions regarding the fungal life cycle. Accordingly a huge number of light-regulated genes is found in fungi^12,13^. With exception of the rhodopsins all fungal photoreceptors react either to blue (WC-1^14^, Vivid^15^, Photolyase^16^, Cryptochrome^17^) or red light (phytochromes^18^). In contrast, fungal rhodopsins perceive green light and their biological function is still under investigation. Recently we noticed that the fungal rhodopsin CarO from *F. fujikuroi* retards the germination of conidia harvested from light-grown mycelia^19^. This green light-regulation controlled by a rhodopsin might play a role during plant infection by *F. fujikuroi* which is a rice pathogen, provoking a frequent and widely spread plant disease, the bakanae^20,21^.

In our previous study, the retarded spore germination was analysed from pictures that were taken at fixed time (12 hours) after germination^19^. Although in this way statistically relevant data were obtained, the dynamics of the early development was not elucidated by this approach. In particular, from these data it could not be deciphered, if only the initiation of conidia germination was retarded or if the complete germination process was slowed down in presence of the active rhodopsin CarO. Such detailed information would become accessible from a time-resolved, computer-assisted germination analysis of individual conidia (single conidia tracking) rather than from real-time monitoring the growth of colonies^22^.

Although powerful tools are available for the analysis of mycelial growth^23–26^, they neither would allow for analysing multiple conidia at the same time with low magnification nor for tracking the temporal area increase during the early germination. Thus, these tools are not optimized for our purpose, which is the automated analysis of *F. fujikuroi* conidia germination. “HyphArea” is a module specialized for the analysis of the distribution of mycelia at distinct time points (2–4 days after inoculation) with growth-adapted magnifications within plant tissue^23^. Another tool is optimized for semiautomatic image analysis using distinct time points for randomized sampling to control the fermentation process of *Trichoderma resii*^24^. In a more recent approach the branching frequency of virtual filamentous microbes was quantified using fractal analysis leading to the development of the ImageJ plugin AnaMorf^25^. Beside the tools that are developed and optimized for the analysis of fungal growth, we also considered tools designed for the analysis of filamentous growth of other cell types like bacteria^27^, plants^28^, and neurons^29^. Especially, for tracking and analysis of neural growth and branching, many different toolboxes/algorithms have been developed recently, and most of them are available as open source software^30–37^. Nevertheless, also these tools are either optimized for the analysis of a single neuron with focus in branching events or they are designed to distinguish cellular features (eg. soma, axon, and dendrites), which do not have a corresponding analogue in fungi.

Thus, inspired by the image processing routines previously described^38^, we developed an implementation for the open source software ImageJ called “HyphaTracker”, allowing for computer-assisted, time-resolved analysis of fungal area extension. HyphaTracker is designed for the analysis of germinating fungal spores and allows for semi-automatic image processing of multiple germlings per field of view. The toolbox enables the exclusion of particles and crossing hyphae during analysis and provides time-resolved data of the area dynamics of fungal germlings. We evaluated the accuracy of HyphaTracker with simulated ground truth data. With the novel toolbox we analysed the germination profile of the conidia of rhodopsin-deficient *F. fujikuroi* strains^39,40^ versus their reference strains and revealed differences in the dynamics of the conidia germination.

## Results

### HyphaTracker – automated image analysis to identify the origin of germlings and to exclude crossing hyphae

For the analysis of the dynamics of conidia germination we implemented an interactive macro for the open source software ImageJ (Fiji)^41^ called HyphaTracker. For reliable data evaluation it was important that the toolbox could distinguish conidia from background and other particles. Furthermore, it should allow to assign an identification number (ID) to each conidium/germling recorded at different time points (frames). Crossing hyphae and hyphae growing out of the image boundary should be excluded from the analysis, provided, that these are rare events. After analysis, the tool should summarize the data in a format that is suitable for further data evaluation in data analysis software.

**Figure 1:**
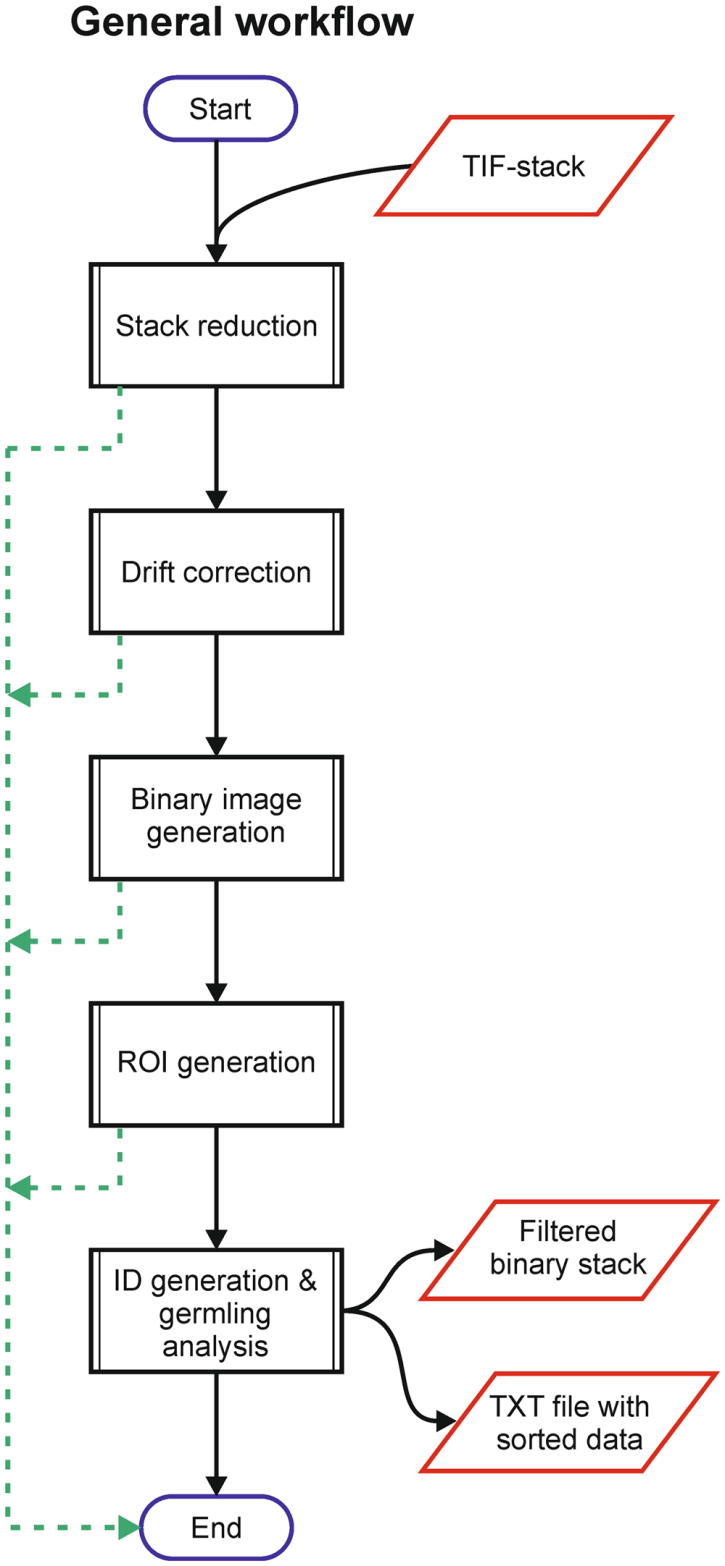
Schematic representation of software-assisted image analysis by the HyphaTracker algorithm (Flowchart). The image time series is recorded in 16-bit resolution in TIF format. Five independent features are available in HyphaTracker for consecutive image processing/analysis: 1. Stack reduction, 2. Drift correction, 3. Binary image generation 4. ROI generation, and 5. GermlingID generation with conidia analysis. The filtered data sorted according to the detected conidia are summarized in a txt-file and optionally as a filtered binary image. For detailed explanation please consult Methods and Supplementary Information.

As summarized in Fig. 1 the HyphaTracker toolbox encompasses 5 routines that are designed to be used sequentially. Nevertheless, every routine can also be run separately, if the appropriate input data is provided (Supplementary Information). The first option allows for reducing the number of images in the time series to a freely eligible reasonable number, to speed up data analysis. Though for video documentation it is worth of acquiring images at high frame rates of e.g. 0.2 min^−1^, it can be oversampled for temporal analysis and the number of images in the series might be reduced without affecting the outcome, as long as the sampling rate is high enough to follow hyphal dynamics^42^ (Supplemental Fig. S7). The second feature encompasses the correction of lateral sample drift that might have occurred during the image recording. Importantly, during data acquisition drift should always be avoided or reduced as axial drift is not corrected by the software.

In the following third part the bit depth of the drift-corrected time series is reduced from 16-bit to binary (See Methods and Supplementary Information 1.3). The routine includes a background reduction via combined “Rolling ball” and Gaussian filter with freely adjustable width (standard radius 20 pixel (px) and 1 px, respectively). Alternative filter settings can be elected by the user depending on the objects to be analysed. The threshold level is automatically chosen by the ImageJ default algorithm, but can be adjusted manually if necessary. Once the image series is binarised HyphaTracker identifies regions of interest (ROIs). In this step initial biological characteristics of the conidia or the young germlings can be used to filter the data i.e. the minimal and maximal circularity as well as the minimal and maximal area of the ROIs can be defined. In the last step these ROIs are analysed and germling IDs are assigned to every ROI while excluding any hyphae crossing other germlings or the boundary of the image. The sorted data are saved as a text file that can be imported into data analysis software thus allowing for the time-resolved analysis of the area increase of single germlings. Optionally also the filtered image series is displayed.

### Evaluation of the HyphaTracker toolbox in terms of accuracy

For testing the performance of HyphaTracker we analysed simulated data resembling the experimental time series in terms of hyphal shape, growth, image size and image signal-to-noise characteristics and compared ground truth with analysis results (for details consult Methods). Each image series contained 20 germlings and the images size was set to 4000 × 4000 pixel corresponding to 2560 × 2560 µm². For the simulation of filamentous growth well defined parameters were chosen. The initial hyphal length was normally distributed with mean of 50 µm and standard deviation of 10 µm. The hyphae were growing for a total time of 60,000 s with a time step of 20 s using a persistence length of 20 µm, a rate constant of 1 * 10^−4^ s^−1^, and an initialization constant of 2 * 10^−2^ resulting in a lag time of 39,318 s. The movie was rendered from images every 1200 s with signal-to-noise ratios (SNRs; mean signal relative to the standard deviation of the background signal) of 13.3 (11.2 dB), 6.7 (8.3 dB), and 3.3 (5.2 dB). Such simulation was repeated 10 times to get sufficient statistics. It should be noted that the movies rendered with different signal-to-noise levels contain the same simulated polymer data.

Analysis by HyphaTracker made sure that only those germlings were considered that do not cross the image boundary or any other germling. Thus, we detected 193, 194 and 193 traces from 200 simulated polymers altogether (from 10 image series for each SNR of 13.3, 6.7 and 3.3, respectively). For each germling we detected traces from 46.9, 46.6 and 46.7 images on average (within movies with 50 images) since some germlings fell out of analysis by crossing the image boundary or other germlings.

**Figure 2:**
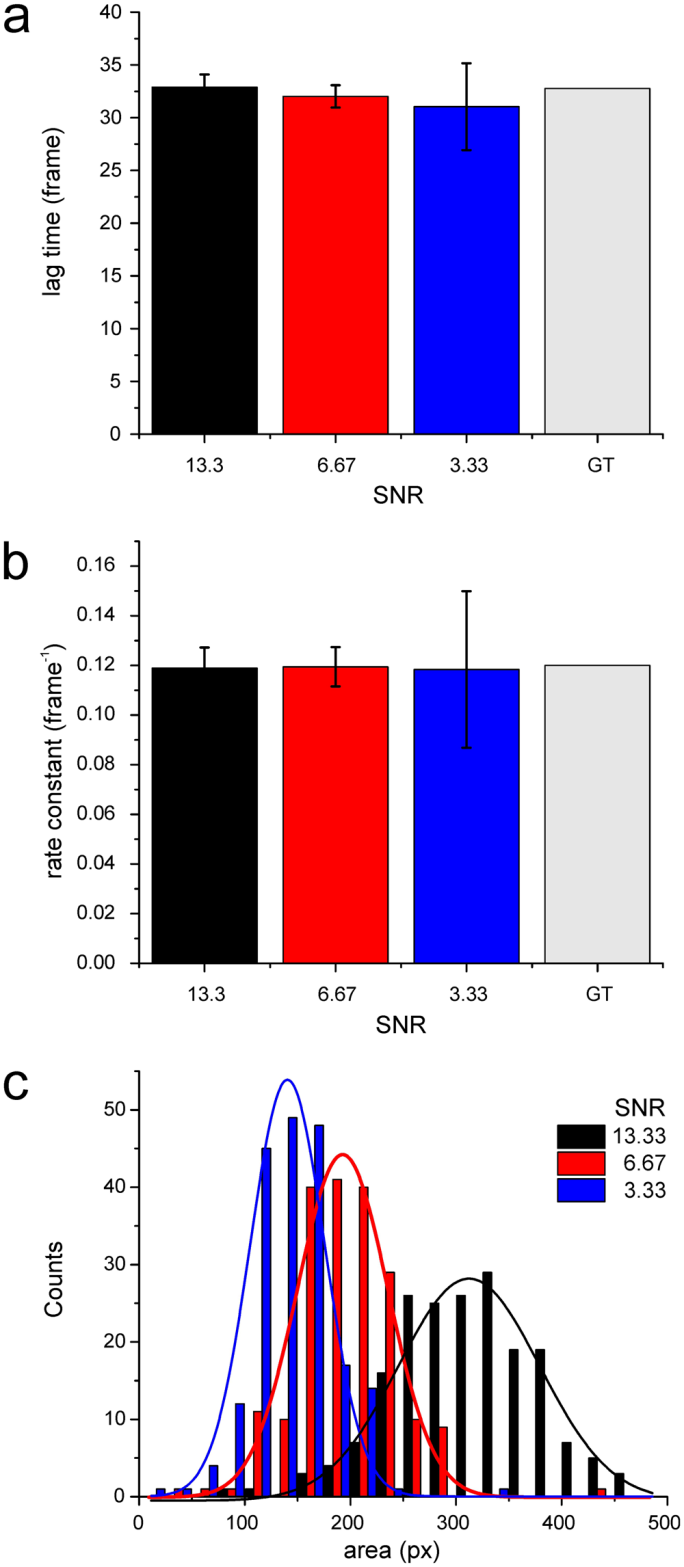
Test for accuracy of the HyphaTracker toolbox on fungal growth simulation. Hyphae were described as two-dimensional worm-like-chains with fixed persistence length and a variable contour length to resemble experimental observations as close as possible. Simulated image series exhibiting different SNRs as indicated were analysed by the HyphaTracker. The time resolved area data were fit using a lag-exponential growth model^52^ and the parameters obtained were compared with ground truth (GT). (**a**) Lag-time. (**b**) Rate constant. (**c**) Initial-area (offset). Note, that the accuracy of HyphaTracker decreased with lower SNRs as indicated by increasing standard deviations (**a**,**b**) and, in comparison to ground truth data, underestimated initial areas (**c**).

When analysing the time dependence of the recorded germling areas (or contour length in the simulation), we found good agreement in terms of an exponential time dependence taking over after a certain lag time (Fig. 2). According to the parameters used for the simulation a lag time of 32.8 frames and a rate constant of 0.12 frame^−1^ were expected to be revealed by HyphaTracker analysis. Indeed, as shown in Fig. 2a,b values for lag time of 32.9 ± 1.2 (SNR 13.3), 32 ± 1.1 (SNR 6.7), 31 ± 4.1 (SNR 3.3) and rate constant of 0.119 ± 0.008 (SNR 13.3), 0.119 ± 0.008 (SNR 6.7), 0.118 ± 0.032 (SNR 3.3) were in good accordance with the ground truth. At low SNRs standard deviation increased while the mean value was only marginally influenced.

The initial size of the conidia could not be directly compared with ground truth, as HyphaTracker follows the evolution of germling areas whereas in the simulation the initial hyphal length was defined. We found the relative distributions of initial areas to follow a Gauss-distribution with a mean value significantly declining with decreasing SNRs (Fig. 2c). This can be explained by the fact that the threshold level used in the “Create binary image” routine has to be increased for lower SNRs to reveal suitable binary images, potentially leading to underestimation of the area. Nevertheless, the hyphal length itself should not or only marginally be influenced by the SNR. Thus, the standard deviation of the initial area is expected to be mainly influenced by the standard deviation of initial length (ground truth 0.2). Accordingly, the relative standard deviation was determined to be 0.24, 0.25, and 0.27 for a SNR of 13.33, 6.67, and 3.33, respectively. In conclusion, the comparison of simulated ground truth data and the results from HyphaTracker confirms that HyphaTracker provides reliable analysis of movies from growing hyphae even at SNRs much worse than encountered in a typical experiment.

### Analysis of *F. fujikuroi* strains by HyphaTracker

**Figure 3:**
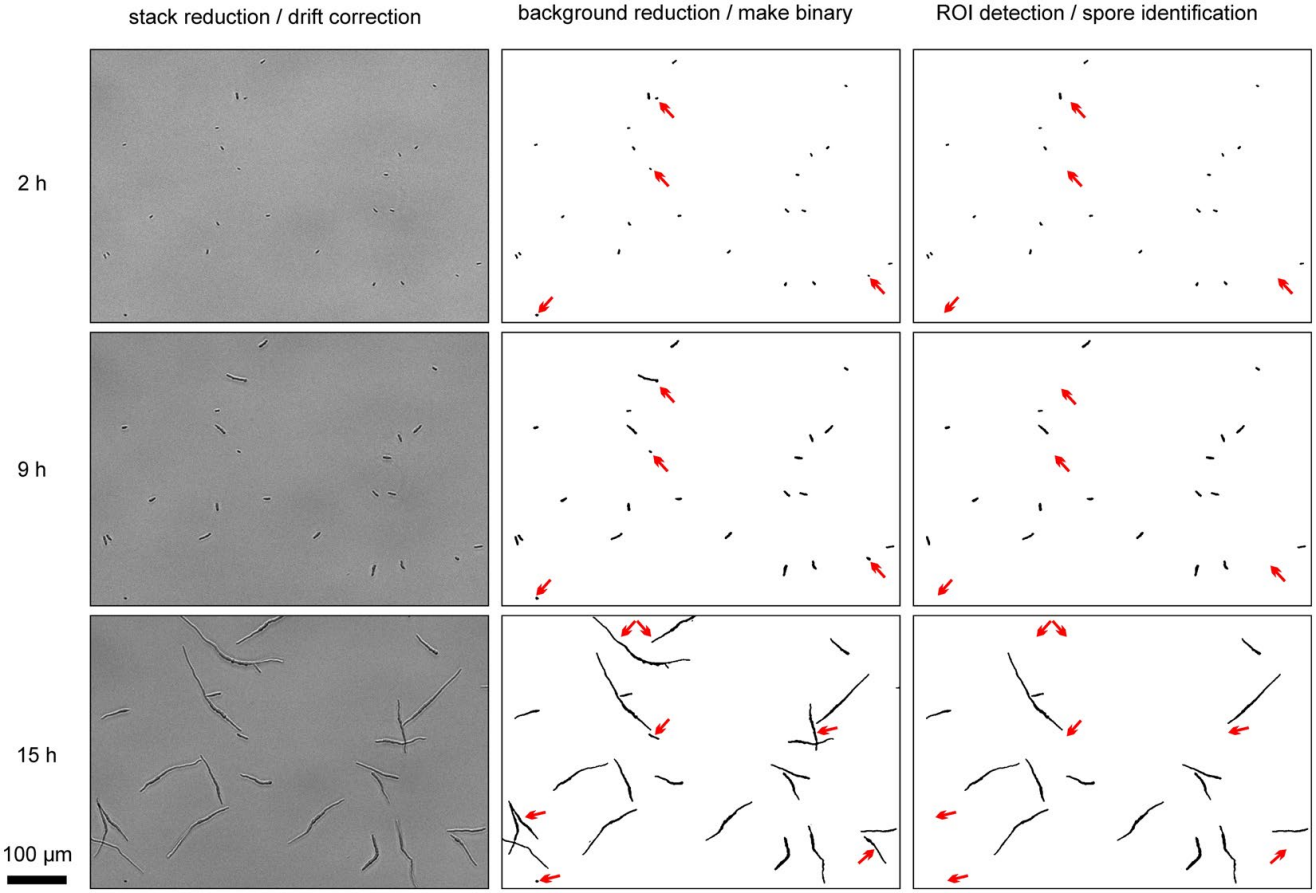
Image processing by HyphaTracker macro for automated data evaluation. Germination of conidia of the CarO-deficient *F. fujikuroi* strain was recorded with a frame rate of 0.2 min^−1^ in a 16-bit image time series (HT-teststack1). Three different time points as indicated are given to visualize the processing procedure of the images by HyphaTracker. In the left column 16-bit images are shown after data reduction and drift correction. The hyphae are slightly out of focus to enable dark appearance in the microscope image. In the middle column the result of step 3 (Binary image generation) of HyphaTracker is shown. In step 4 and 5 (right column) the ROIs are identified and filtered for their size and eventually occurring crossing events or contact to the edge. Red arrows highlight such germlings that were automatically removed from the analysis. Black bar represents 100 µm.

Hence, we used HyphaTracker to analyse the germination dynamics of four different *F. fujikuroi* strains, the FKMC1995 wild type, the Δ*opsA* strain SF223^40^, the CarO-deficient strain SF100, and its control SF101^39^. The image time series comprised the germination of conidia during >15 hours. As a requirement we optimized in a first instance the microscopic protocols for the subsequent automatic evaluation. Conidia were seeded in Poly-D-Lysine coated glass surfaces to avoid streaming and displacement of conidia during the image recording process. Furthermore, all optical components were thoroughly cleaned as requirement for generating low-noise background and avoiding false-positive localisation. In addition, we realized that the slightly defocused recording of images eased the conversion to binary images and thus the detection of hyphae. By this method we gained a strong contrast between the dark hyphae and the bright background. Taking these improvements into account we succeeded in generating suitable image time series (Fig. 3). Note, that computational analysis with HyphaTracker of image-series recorded in the focal plane will require adapted pre-processing of the recorded images before binarisation. In accordance, using a median filter (radius 3 px) for background filtering we yielded satisfying results in the time-resolved analysis of the germination of *Lichtheimia cromybyfera* conidia that were imaged in the focal plane. The aspects of those initial experiments will be analysed in detail in another project.

The complete analysis took between a few minutes to maximum 40 min depending on the computational capacity of the PC system, the size of the time series, and the amount of conidia to be analysed during the routine. For our dynamics analysis we reduced the frame rate from 12 h^−1^ to 3 h^−1^ to diminish the data volume while keeping sufficient information for the analysis of germination dynamics (Supplemental Fig. S7). The drift correction was very important for gaining convenient germling identifications and filtering of false-positive events. As this routine is very fast (<1 min) it is highly recommended to run drift-correction if the setup is not completely drift-free. However, displaced conidia, or conidia dramatically changing their morphology as well as hyphae growing out of the z-focus into the liquid are not sufficiently interpreted and thus were manually excluded from the analysis to reduce the risk for misinterpretation of the data. Furthermore, the germling identification was noticed to react very sensitive to the radius chosen around the reference conidium (Supplementary Information Fig. S11). In general, 80–85% of the crossing hyphae were automatically rejected from the analysis while the hyphae crossing the border were completely separated from data analysis. In rare events, the crossing hyphae were not detected especially when more than two hyphae were crossing. Some conidia are still suspended when image acquisition starts and reach the focal plane later in the image series. As a consequence, the initial conidium might be lacking in the reference frame for the GermlingID assignment leading to erroneous filtering. This problem could be avoided when a relatively late reference frame was chosen that includes all later germlings (Supplementary Information Fig. S10). Furthermore, the spore density should be balanced in a way that as many germination events as possible can be recorded simultaneously, but that the percentage of crossing events is kept low. In conclusion, manual control of the automatically generated data is inevitable to reduce the risk for misinterpretation of the data. However, overall the analysis routine was working fine as depicted in Fig. 3.

### The computed analysis by HyphaTracker of fungal growth reveals earlier germination of the CarO-deficient strain

**Figure 4:**
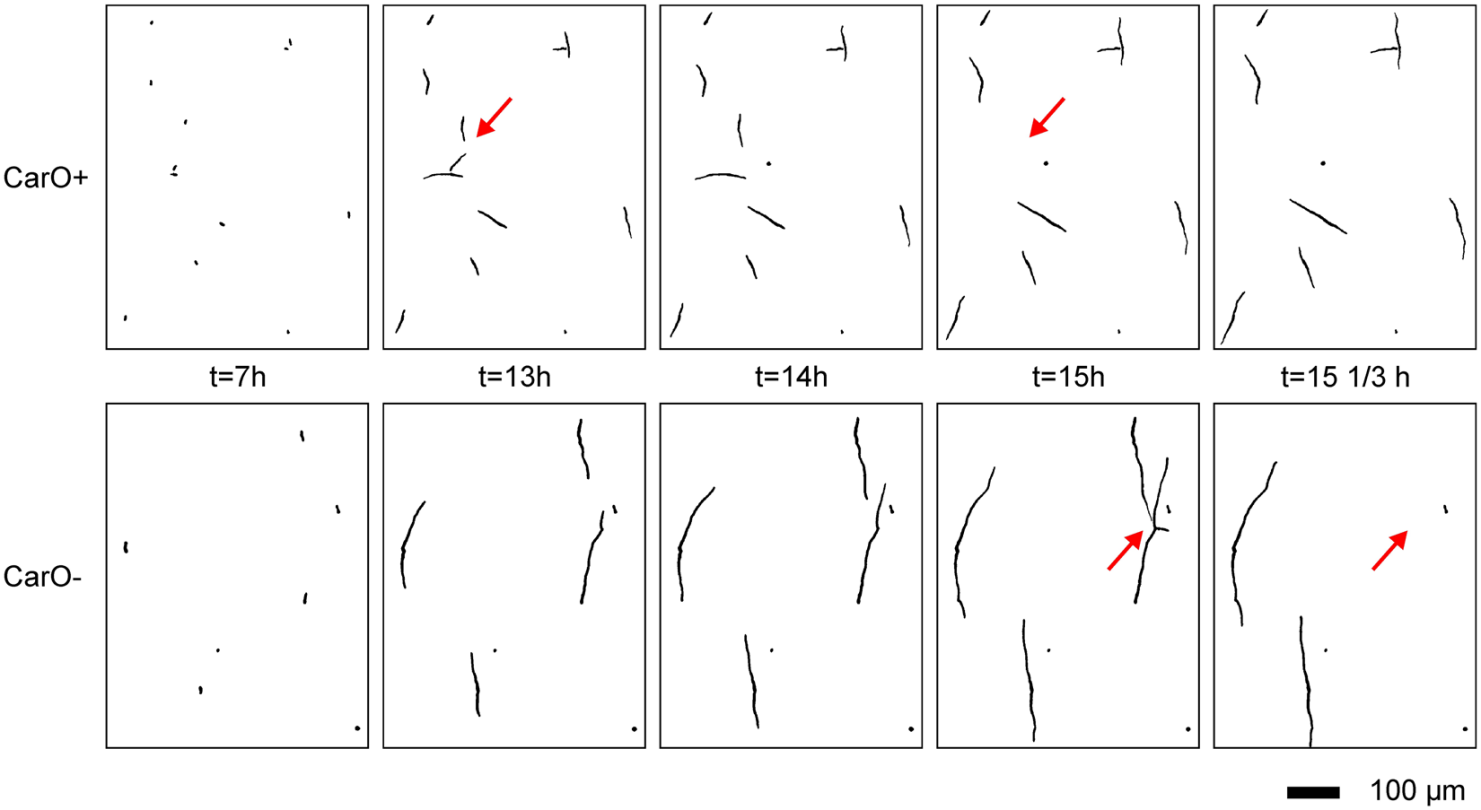
Comparison of the time course of germination of *F. fujikuroi* CarO+ and CarO− strain. Binary images converted and filtered by HyphaTracker macro are shown. Red arrows are indicating conidia that are crossing during growth and were automatically separated from the analysis. Germlings of the rhodopsin-expressing strain exhibit a decreased growth after 7, 12, 14, 15, and 15 1/3 hours compared to the rhodopsin-deficient strain. Black bar represents 100 µm.

The fungal rhodopsin CarO is thought to be involved in the regulation of conidia germination in *F. fujikuroi*^19^. In accordance, in a time-resolved microscopic analysis we observed faster hyphal extension of the CarO-deficient strain compared to its rhodopsin-expressing control strain (Fig. 4). In our previous investigation we showed pronounced differences in the hyphal length after 12 hours. However, since manual analysis of conidia germination dynamics is very time-consuming, we did not yet investigate the kinetics of the retarded germination of the CarO^+^ strain (in comparison to CarO−).

**Figure 5:**
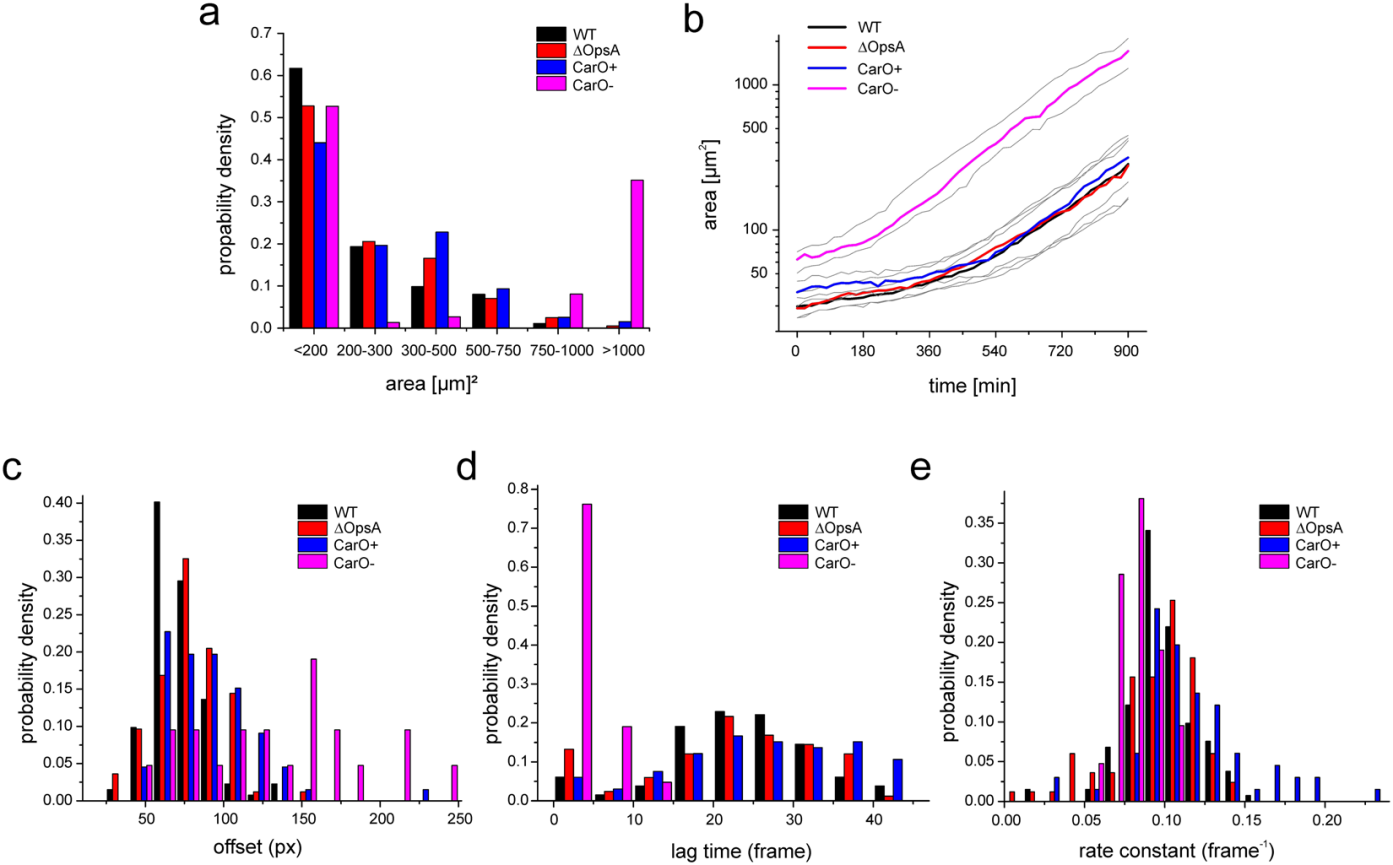
Germination behaviour of *F. fujikuroi* conidia analysed by the HyphaTracker toolbox. (**a**) Histogram of the probability density of germling areas after 15 hours incubation time. Note, that all strains with exception of the CarO-deficient strain exhibit similar germination profile in this histogram. In contrast the rhodopsin deficient mutant exhibits a much higher number of hyphae with areas >750 µm^2^. (**b**) Hyphae areas generated with HyphaTracker plotted as function of time. The lines represent median (coloured for wild type, ΔOpsA, CarO+, and CarO− as indicated) and quartiles (gray) for each dataset (wild-type: n = 133 in 5 independent experiments), ΔOpsA: n = 83, 4 exp., CarO+: n = 67, 3 exp., CarO−: n = 21, 9 exp.). Obviously, germination of the CarO-deficient strain starts earlier and growth occurs faster than in the CarO^+^ strain, wild type, and the OpsA deletion mutant (**c**–**e**). Probability density functions as estimated by fitting experimental data using the Baranyi model. Histograms are shown for offset (initial area; **c**), lag time (**d**) and rate constant (**e**) for wild type, ΔOpsA, CarO+, and CarO− in black, red, blue, and magenta, respectively.

In accordance with our previous study, the analysis by HyphaTracker revealed higher extension of hyphae that were deficient in CarO expression (Fig. 5a). In contrast the ΔOpsA mutant and the wild type control did not exhibit remarkable differences compared to the CarO^+^ strain, suggesting a non-crucial role of the rhodopsin OpsA during spore germination. After 15 h the majority of the germlings of wt (n = 308, 5 exp.), CarO^+^ (n = 193, 3 exp.), and ΔOpsA (n = 199, 4 exp.) strains exhibited surfaces <300 µm^2^, while the CarO deficient strain (n = 100 in 9 exp.) exhibited strongly increased hyphal areas with >95% of hyphae >300 µm^2^.

We aimed to reveal, if in the CarO-deficient strain, in comparison to the other strains, the germination starts earlier or if the growth velocity is enhanced. Therefore, we performed dynamic analysis of conidia germination with HyphaTracker using a time-resolved image series with a reduced frame rate of 3 h^−1^) resulting in experimental data on the time-dependent sizes of growing hyphae.

Plotting areas over time (i.e. frame number) revealed an accelerated growth with some variation between individual hyphae (Fig. 5b, Supplementary Fig. S12). Due to the faster extension of the CarO-deficient strain the amount of conidia of this strain had to be reduced in comparison to the other strains to avoid frequent crossing of germlings. As a prerequisite the germination behaviour was independent of the conidia concentration used during the experiment in the range between 2,000 and 5,000 conidia/µl (Supplementary Fig. S13) suggesting no or only minor effects of quorum sensing known from other ascomycetes^43,44^ under these conditions. Nevertheless, we had to repeat the experiment with CarO deficient mutant several times in order to gain sufficient amount of data (CarO−: 21 germlings/9 exp., CarO^+^: 67 germlings/3 exp.; wild-type: 133 germlings/5 exp., ΔOpsA: 83 germlings/4 exp.). As strict confinement, for all strains only germlings at least doubling their area during the experimental time were included in the data analysis.

The time dependence of measured areas *a*_*i*_*(t)* could be well described by a growth model that incorporates a lag time and an exponential growth. We used the Baranyi model Eq. 5 with initialization factor *q*, an offset *o*_*i*_ representing initial hypha size, and rate constant *k*_*i*_ to fit all data curves and compared the extracted parameter distributions.

All extracted values for the parameters offset, lag time (as computed from q and k following Eq. 4) and rate constant appear to be Gauss-distributed (Fig. 5c–e; comparison of mean and standard deviation is given in Table 1). In the logarithmic plot of areas (Fig. 5b) it appears that the hyphae of the CarO-deficient strain grow with a – in comparison to its control strain – reduced lag time therefore reaching larger sizes in the given observation time. The fit parameter for CarO− indeed reflect this behaviour (Table 1), as we found a significant increase in offset (student’s t-test, p < 0.005), a strong decrease in lag time (student’s t-test, p < 0.001), and a slight decrease in rate constant (student’s t-test, p < 0.001) indicating accelerated growth right from the starting point of the measurements. In contrast, high similarity was found between parameters from wild type and ∆OpsA strains (Table 1).

**Table 1:** Growth characteristics of various *F. fujikuroi* strains.

Sample	Fungal strain	offset *o*_*i*_ (initial conidia/hypha size)	lag time *t*_*lag*_ (time when fungal growth enters exponential phase)	rate constant *k* (rate constant of exponential growth)	n
wild type	FKMC1995 *Fusarium fujikuroi* (*G. fujikuroi* mating population C) Kansas State University Collection (Manhattan, KS)	72 (17) px/29.3 (6.9) µm^2^	24 (10) frame/480 (200) min	0.10 (0.02) frame^−1^/0.005 (0.001) min^−1^	123
∆OpsA	Mutant FKMC1995 strain with deleted rhodopsin gene *opsA*^40^ (SF223)	79 (21) px/32.2 (8.6) µm^2^	22 (11) frame/440 (220) min	0.10 (0.03) frame^−1^/0.005 (0.0015) min^−1^	83
CarO+	Mutant FKMC1995 strain with intact rhodopsin gene *carO* used as isogenic reference for CarO− strain^39^ (SF101)	98 (66) px/39.9 (26.9) µm^2^	26 (11) frame/520 (220) min	0.12 (0.03) frame^−1^/0.006 (0.0015) min^−1^	66
CarO−	Mutant FKMC1995 strain with disrupted rhodopsin gene *carO* (ORF frame shift)^39^ (SF100)	133 (53) px/54.1 (21.6) µm^2^	2 (4) frame/40 (80) min	0.08 (0.01) frame^−1^/0.004 (0.0005) min^−1^	21

Mean and standard deviation of estimated normal distributions from n data points representing offset, lag time and rate constant.

## Discussion

Fungal development starts from conidia or sexual spores that represent essential elements in the fungal life cycle. They enable the efficient dispersal of the fungus and ensure its survival under unfavourable conditions. For the fungal spore it is only worth of germinating when its environment provides convenient growth conditions and sufficient nutrients to ensure the development of a mycelium. Therefore, the germination process in fungi is highly regulated by ambient conditions^45^. Especially light is as an important trigger influencing the regulation of spore germination in various fungal species^11,19,46,47^. A more detailed knowledge of the germination processes would, in the long-perspective, allow the development of strategies to reduce fungal contaminations and/or to optimize fungal colonisation in bioprocessing. Thus, experimental approaches gaining data about the dynamics of germination are in a constant demand.

Microscopic analysis provides a convenient method for the qualitative analysis of germination dynamics. However, manual evaluation of suchlike data is extremely time-consuming especially when statistically relevant data are required. Thus, image time series should be analysed by suitable computational software enabling automated or semi-automated image analysis. Though there are several computational tools for the analysis of filamentous growth of fungi^23–26^ and neurites^30–37^ available, to our best knowledge none of the available tools is intended for the analysis of the early hyphal development of multiple germlings at the same time. In general, many available analytical tools are designed for qualitative analysis of filamentous growth rather than quantitative analysis of multiple germling-events in parallel^23–25^. In these approaches cells are imaged at high magnifications (40–100-fold) to ensure the recording of many cellular details.

In the present study we introduce a novel toolbox implemented in ImageJ called HyphaTracker, which is designed for the quantitative analysis of early germination by imaging 20–100 conidia at the same time while using a low magnification. The novel toolbox was designed to adapt the raw images for data analysis (image processing) and to report the area of every germling at each time point (germling identification) while omitting crossing hyphae and other objects from the data analysis. Omitting crossing objects is only admissible if the density of objects is low and the crossing events are seldom in comparison to the number of objects analysed. In the simulated data the percentage of removed hyphae was low <5% for all SNRs. Nevertheless, crossing events are a common issue while tracking multiple objects simultaneously and omitting them is a suitable solution to avoid misinterpretation of quantitative data^28^. In the long term view approaches of disentangling filamentous networks into single filaments while preserving branches might be implemented^48^.

HyphaTracker is conveniently controlled by GUI and enables fast automated data analysis of many germlings in parallel according to customized settings. HyphaTracker provides a filtered output of the temporal area increase of multiple single conidia from the same batch. Beyond that, the filtered binary output provides the fundament for further detailed customized analysis of the time series. By that, the HyphaTracker output might also help to reduce erroneous detection by other previously described tools. For example the resulting binarised time series, filtered by the HyphaTracker might be further analysed by the ImageJ plugin AnalyzeSkeleton^49^ to detect temporal branching events in the developing fungus and to estimate the actual length of the filtered germlings. In general, such further analytical steps could be easily integrated into the source code of HyphaTracker.

Based on the simulated data used for ground truth analysis we identified the filtering routine of HyphaTracker to be robust and reliable. The parameters describing the kinetics of hyphal development were revealed with high accuracy even at very low SNRs (Fig. 2). Nevertheless, high optical contrast of the fungi to their environment eases the detection of germlings by the HyphaTracker tool due to higher SNR. We empirically chose a slightly defocused setting leading to dark appearance of conidia and germinating hyphae in the bright-light microscope. Hence, the area of the hyphae and conidia might be slightly overestimated, but on the other hand the generation of binary images might slightly reduce the area in the edges of the fungi. HyphaTracker also enables the analysis of germlings imaged in the focal plane presupposed adequate image pre-processing. A general challenge is the phenomenon that in the bright-field microscopy often hyphae appear paler in their centre, leading to their appearance as holed structures in the binary image. Therefore, the ImageJ binary filter “Fill holes” was implemented into the HyphaTracker code as part of the “Detect germling” routine recovering holes in the hyphae. It should be noted that the object labelling does not take into account imaging artefacts. Thus, it is important to review the data manually or to define confinements in the meta-analysis that are restrictive for hyphae. This is especially the case for analyses in which conidia appear later in the image series or for hyphae that grow out of the focal plane (See also Supplementary Information).

To test the accuracy of HyphaTracker we used simulated data containing ground truth information. For the simulation we aimed to formulate a relative realistic scenario. There are various investigations dealing with the dynamics of conidia germination in fungi. Most of them focus on the complete mycelial growth with duration of several days without showing a detailed extension in the first hours^1,5^, only showing the percentage of germination over time and growth rate^50^, using different supporting substrates^3^ or focussing in germination events^51^. However, to the best of our knowledge there are no studies analysing in detail the dynamics of germtube formation and early hyphal development. A general aspect in the growth kinetics of individual hyphae is the exponential hyphal extension at a constant specific rate in the early growth phase^2^. The Baranyi model^52^ used for the analysis of the germination data is a lag-exponential growth model, originally introduced for modelling bacterial growth in food but also valid for modelling growth in fungi^4^. This behaviour is described in detail by Gougouli *et al*.^1^ and Prosser^2^: The hyphal extension starts with conidia germination and germ-tube formation. The extension rate accelerates while the germ-tube length increases leading to a self-supporting growth. This growth behaviour can be described by an exponential growth with constant specific rate. Eventually extension rate reaches a constant value leading to a linear extension of the hypha most likely due to limitation by the transport of material from the tip to regions behind. The growth rate of fungal hyphae is an important measure for the effect of ambient factors on fungal growth^3–5^.

Recently we reported that the green-light perceiving rhodopsin CarO retards the early hyphal development in *F. fujikuroi*^19^. However, due to the absence of suitable methods the kinetics of germination was not investigated and we only analysed the statistical length distribution after 12 hours of germlings that were measured manually. Now, the HyphaTracker toolbox allowed us to analyse the area extension in the first 15 hours of 4 different *F. fujikuroi* strains, two rhodopsin deficient (Δ*opsA*, CarO−) and two rhodopsin expressing (wild type, CarO+) control strains (Table 1). Only the CarO-deficient strain showed a clearly accelerated and earlier germination pattern, while the other three strains exhibited similar, but slower kinetics under the tested conditions (Fig. 5). Using the Baranyi model^52^ (Eq. 5) we deduced a clearly reduced lag-time of 40 min (frame 2) when compared with the other strains that were in the range of 7–9 hours (frame 22 to 26)). Accordingly, in the end of the measurement a higher number of elongated germlings is found from the CarO-deficient strain (Fig. 4a). This is also in accordance with our previous, non-dynamical data of germlings-length manually measured after 12 hours growth^19^. On the other hand, the rate constant was slightly reduced in the CarO-deficient strain. The less pronounced exponential growth indicated by the slightly reduced rate constant in the CarO-deficient strain might explain the previous finding that the growth pattern does not differ between CarO+ and CarO− strain when compared in the colony level^19^. Interestingly, linear growth phase was already entered by fast growing hyphae of CarO− in our experiments (Supplementary Fig. S12).

It is a puzzling question why the absence of the fungal rhodopsin CarO leads to altered kinetics. One may speculate that physiological processes connected with the germination as described for the related species *F. graminearum*^53^ might be triggered much earlier and to a greater extent when the rhodopsin CarO is not present. The moderate area extension in the initial lag phase can presumably be correlated with the isotropic extension of the conidia which involves conidia swelling and reorientation of cell wall^54,55^, while the later exponential increase is connected with anisotropic growth (growth of germ tube)^55^. CarO was characterised as an outwardly acting, light-gated proton pump. Though a proton pump in principle is capable of influencing the pH during the germination, we would expect the pump activity to support the environmental acidification and by that fungal germination, which is in contrast to our findings. Thus, it might be considered that the processing of the CarO signal requires some time-consuming response of the conidium that becomes obsolete in the absence of the rhodopsin leading to a drastically reduced lag time. Further experiments are planned in the near future to understand the role of CarO as a regulator in conidia germination. In this respect, also the potential effect of weak organic acids and auxins on the germination dynamics of *F. fujikuroi* are of interest.

## Conclusion

HyphaTracker allows for computer-assisted analysis of time-resolved microscopic recordings of the conidia germination revealing valuable data in the early germination dynamics. The HyphaTracker toolbox provides great potential for analysis of mutant strains where physiologically relevant proteins are knocked down. Thus, the dynamic analysis of early germination under varying treatments and conditions might provide novel information on the physiological response of fungi and thus helps for the development of new antifungal agents. The toolbox also allows for following the germination dynamics of conidia after exposure to various ambient conditions^56–58^ and plant exudates^59^ like recently done with other *Fusarium* species. Data gained by such analyses are very important, as avoidance of fungal germination is a convenient strategy to protect goods from destruction by fungi. Though developed for the ascomycete *F. fujikuroi* the toolbox is also suitable for other fungi. Furthermore, due to open source implementation in ImageJ HyphaTracker might additionally be adapted by their users to their specific requirement. If strongly fluorescent fungal strains with homogenously distributed fluorophores are available, we expect them to be also applicable to HyphaTracker.

## Methods

### Cultivation of fungi

*Fusarium fujikuroi* strains were kindly provided by J.F. Leslie (Kansas State University Collection, Manhattan, USA) and J. Avalos (University of Seville, Spain). We used the wild type strain FKMC1995, the Δ*opsA* mutant strain SF223^40^, and the *carO* deficient mutant strain SF100 together with its corresponding control strain SF101^39^. The strains were grown 7–9 days in DG_Asn_ minimal media^60^ at 28 °C in light at a light irradiance of ~7 mW cm^−^². Conidia were harvested from the mycelia in sterile water by scraping. Mycelial parts were removed by filtering the conidia suspension through pore-size 2 glass filters (Robu, Hattert, Germany). The conidia suspension was centrifuged (5 min, 2000 g, 4 °C), and the pellet was washed with sterile H_2_O. Conidia were resuspended in H_2_O and counted.

### Germination experiments and image acquisition

Microscopic investigation was performed in micro chambers mounted on glass coverslips (Labtek II, Thermo-Scientific, Braunschweig, Germany). Glass surface was etched in 0.5 M NaOH for 2 hours and coated with 0.01% Poly-D-Lysine (P7886, Sigma, Taufkirchen, Germany) for at least 4 hours at room temperature. After etching and coating procedure, the wells were washed thoroughly three times with sterile H_2_O and filled with 500 µl DG-medium. Conidia were spread in a density of 2,000–5,000 conidia/well. One may at this point add suitable fiducial markers in order to ensure the success of the drift correction method. Conidia germination was photo-documented by means of an inverted microscope (Axiovert200, Zeiss, Germany) using an objective with 10-fold magnification (NA 0.3 Plan-Neofluar, Zeiss). The chamber was warmed to 28 °C by means of a custom-made warming entity. Time series of conidia germination were recorded with a sCMOS camera (Zyla 5.5, Andor, Belfast, UK; 0.638 µm/px after final magnification) at a frame rate of 0.2 frame min^−1^ for 15 hours. In all experiments the sample was illuminated using the bright field light source with an intensity of 2 mW cm^−^². The focal plane was adjusted in a way that the conidia appeared dark in the bright field image, which eased successful image processing. Hardware was controlled by means of the software µManager^61^ (University of California, San Francisco, USA).

### Image analysis with HyphaTracker

Images were processed using the open source software ImageJ (v1.51g-v1.51n; Fiji package^41^). An algorithm called “HyphaTracker” (Supplementary Software) was designed for the automated detection and analysis of conidia germination (Fig. 1). The toolbox encompasses 5 independent steps that are auxiliary for the automated determination of area incorporated by conidia/germlings, i.e. 1. Data reduction, 2. Drift correction, 3. Binary image creation, 4. ROI generation, and 5. ID generation and germling analysis. All processes can be sequentially performed or used as standalone feature. In the following the settings we used for analysing our data are given. The default settings were tested with regard to the pixel size of our microscopic setup and should be adapted if necessary. For explanation of the general use of the HyphaTracker we refer the reader to the Supplementary Information.

#### Stack reduction

The conidia germination was recorded with a frame rate of 12 frames h^−1^ in recordings lasting over >15 hours (time series of 184 images). In order to optimize the computational performance, we reduced the data resolution. For the data presented in Fig. 5, we reduced the number of analysed frames to 46 using only every 4^th^ image for further analysis.

#### Drift correction

This feature was important, as in the germling analysis ROIs in the first and last frame must correspond to each other and thus the lateral position of the conidium is essential for the determination of the germling ID and exclusion of crossing events. To perform the drift correction an immobile reference point was needed as fiducial marker. We used non-germinated spores or cell debris, immobilized on the glass surface as a reference for the drift correction. The suitability of the particle for drift correction could be justified by the coordinate trajectory. It was required to crop a new section from the corrected time series which was used for further analysis.

#### Create binary image

The time series was recorded with 16-bit resolution. In this routine a binary image time series was generated that was suitable for further analysis. First the background was filtered to reduce the noise within the picture without losing information on the conidia or their germlings. This was done in a combination of rolling ball (20 px) and Gaussian filter (1 px). Then, a threshold was applied to generate the binary image. The threshold level was either determined automatically by the ImageJ default algorithm (based on the isodata^62^ algorithm) or adapted manually in order to separate the germlings from the background signal (See also Supplementary Information, section 1.3). Remaining noise in the binary image was removed via the “Despeckle” function included in ImageJ. “Despeckle” is a median filter that replaces each pixel with the median value in its 3 × 3 neighbourhood. Finally, every spore and hypha surpassing the threshold appeared clearly separated from the white ground.

#### ROI generation

ROIs were generated by the “Analyze Particles” function in ImageJ. Here, objects were filtered for circularity and the area (min. circularity 0.00–0.10, max. circularity 1.00; min area 20 px, max area 100,000 - Infinite). *F. fujikuroi* exhibits conidia with strong polarity thus circular structures are unlikely to present fungal spores. To avoid ROIs not belonging to conidia or germlings (remaining noise signal), the minimal size was set to at least 20 px. This was in accordance with the min value of non-germinated spores of *F. fujikuroi* (7.67 µm^2^; n = 104).

#### GermlingID and analysis

The ROIs were analysed and sorted using three steps, that is the i) determination of ROI origins, ii) determination of master-ROIs, and iii) assigning IDs to ROIs. For the determination of ROI origins, a late reference frame was defined in which all non-germinated spores are visible. From these ROIs coordinates are extracted that serve as origins. Then master-ROIs were determined, i.e. ROIs in the last frame. These ROIs were verified by checking that they only have a single spore origin. Finally, all ROIs were assigned to a master-ROI, resulting in different integer for each master-ROI (ID). Eventually occurring holes within the germling shape are recovered during the analysis by the ImageJ binary filter “fill holes”.

Finally, a text-file was outputted stating area, temporal information (number of frame), and ID along with additional geometrical information of all ROIs. These data were then further analysed using OriginPro2016G (OriginLab Corporation, Northampton, USA) or Mathematica (Wolfram Inc., Version 11.1). Furthermore, a filtered TIF-file was outputted as binary time series that displayed the growth of each germling.

### Source availability

HyphaTracker is provided as Supplementary Software. Updates will be available from http://bcp.phys.strath.ac.uk/photophysics/super-resolution/software/.

### Simulation and ground truth analysis

For testing the performance of HyphaTracker we analysed simulated data that resembles the experimental movies in terms of hypha-shape, growths, image size and image SNR characteristics and compared ground truth with analysis results. All simulations were performed with custom-made code written in Mathematica (Wolfram Inc., Version 11.1). Growing hyphae were assumed to resemble 2-dimensional polymers described as worm-like-chains with a fixed persistence length and a variable contour length. Growth was simulated by sampling the increase of polymers at a fixed time step from a probability distribution representing a certain growth model. It was always confirmed that the step size is smaller than the persistence length^63^. The growth direction varied randomly with a small angle variation Ѳ drawn from the probability density function

1 $${\rm{P}}({\rm{\theta }})=\sqrt{\frac{{L}_{P}}{2\pi \,l}}{e}^{-\frac{{L}_{P}{\theta }^{2}}{2l}},$$

where *L*_*p*_ is the persistence length and *l* is the step size. All parameters and the growth model were chosen in a way to resemble experimental observations as close as possible. In the presented data we assumed a growth model relating to the lag-exponential growth model as stated by Baranyi *et al*.^52^. Here, the length increase ΔL_*C*_ at each time step Δt depends on the absolute time t = *n*Δt (with n being a time step index) and is proportional to the current contour length L_*C*_(*n*Δt), a rate constant *k*, and an adjustment factor *α*(*n*Δt) accounting for a lag time that delays growth:

2 $${{\rm{\Delta }}L}_{c}(n{\rm{\Delta }}t\,)=\alpha (n{\rm{\Delta }}t)\,k\,{{\rm{L}}}_{c}(n{\rm{\Delta }}t){\rm{\Delta }}t,$$

The adjustment factor *α*(*n*Δt) incorporates the idea that a critical substance must accumulate and is characterized by an initialization constant q and the rate constant *k*:

3 $$\alpha (n{\rm{\Delta }}t)=\frac{q}{q+{e}^{-kn{\rm{\Delta }}t}}\,.$$

In this approach a lag time t_*lag*_ is defined as

4 $${t}_{lag}=\frac{\mathrm{ln}(1+1/q)}{k}.$$

In the simulations we varied the initial size L_0*i*_ (with i referring to the individual polymer) of the polymers according to a normal distribution.

From simulated polymer traces a movie was generated by taking a random initial position and orientation for each of 20 polymers on a square of 2560 × 2560 µm² and rasterizing each frame into a binary image with 4000 × 4000 pixel. The images were blurred by a Gaussian filter with radius of 8 pixel to increase the width of the polymer lines. Background was added following the background distribution of experimental images (indicating Gauss distributed intensity values with a standard deviation of 0.03 around a mean of 0.5) and signal to noise was adjusted by changing the average signal strength in relation to mean background values.

For any choice of initial polymer size L_0_, rate constant k, and initialization constant q the contour length L_*c*_(*t*) can be fitted to the analytical expression

5 $$y(t)={{\rm{y}}}_{0}+{\rm{k}}(t-\frac{1}{{\rm{k}}}\,\mathrm{ln}(\frac{1+q}{q+{e}^{-{\rm{k}}t}}))\,$$

with y(*t*) = ln(L_*c*_(*t*)) and y_0_ = ln(L_0_). This way HyphaTracker results could be fitted and the extracted parameters could directly be compared to the simulation inputs.

### Analysis of germination velocity

For the temporal analysis of germination only conidia were used that were present in every frame during the whole observation time. Only conidia showing clear germination (final area after 15 hours at least 2 times the initial area) were used in this evaluation. The image time series was reduced and every 4th image was kept. After processing the data with HyphaTracker the area of each germling was plotted against the time and data were fit using Eq. 5. For better visualisation the median value and the quartile of all conidia of each strain was calculated (Fig. 5a).

### Data availability

All relevant data are included in the manuscript and the supplementary information. The source code of HyphaTracker is provided as supplementary file “HyphaTracker_v1.0.zip”. Updates will be available from http://bcp.phys.strath.ac.uk/photophysics/super-resolution/software/. In addition, various test time series are provided giving the reader the opportunity to test the macro directly in ImageJ. Four full size image time series (2 GB each) are available at https://go.uniwue.de/hyphatracker and more time series can be obtained from the corresponding author upon reasonable request.

## Electronic supplementary material

Supplementary Dataset 2

Supplementary Dataset 1

Supplementary Information
