# Supplementary figures and images for: HyphaTracker: An ImageJ toolbox for time-resolved analysis of spore germination in filamentous fungi
Source: Sci Rep. 2018 Jan 12;8:605. doi: 10.1038/s41598-017-19103-1 (PMC5766585; doi:10.1038/s41598-017-19103-1)

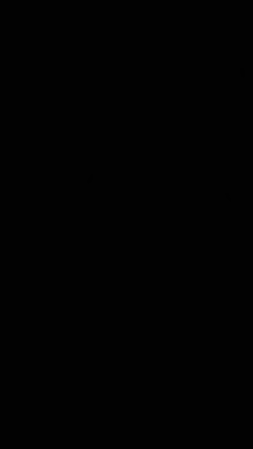

Supplement: Supplementary file 1 — Supplementary Dataset 2 [file 41598_2017_19103_MOESM1_ESM.zip › teststack.tif]
