# Supplementary material for: HyphaTracker: An ImageJ toolbox for time-resolved analysis of spore germination in filamentous fungi
Source: Sci Rep. 2018 Jan 12;8:605. doi: 10.1038/s41598-017-19103-1 (PMC5766585; doi:10.1038/s41598-017-19103-1)
Supplement: Supplementary file 2 — Supplementary Dataset 1 [file 41598_2017_19103_MOESM2_ESM.zip › HyphaTracker_InstallationGuide.pdf]

HyphaTracker 1.0

Installation Guide

A) Download and install ImageJ or Fiji (http://imagej.nih.gov/ij/ or http://fiji.sc/Fiji), update to version 1.51n or higher.

B) Install HyphaTracker by either copying 'HyphaTracker_v1.0.ijm' into the plugins folder of ImageJ/Fiji (suggestion: 'Fiji.app\plugins\Macros') or by selecting *Plugins>Install…* (**Figure 1** (1)). Restart ImageJ/Fiji. Once ImageJ is launched, the macro now appears in *Plugins>Macros>HyphaTracker_v1.0* (2).

C) A keyboard shortcut to HyphaTracker can be assigned using *Plugins>Shortcuts>Add Shortcut…*

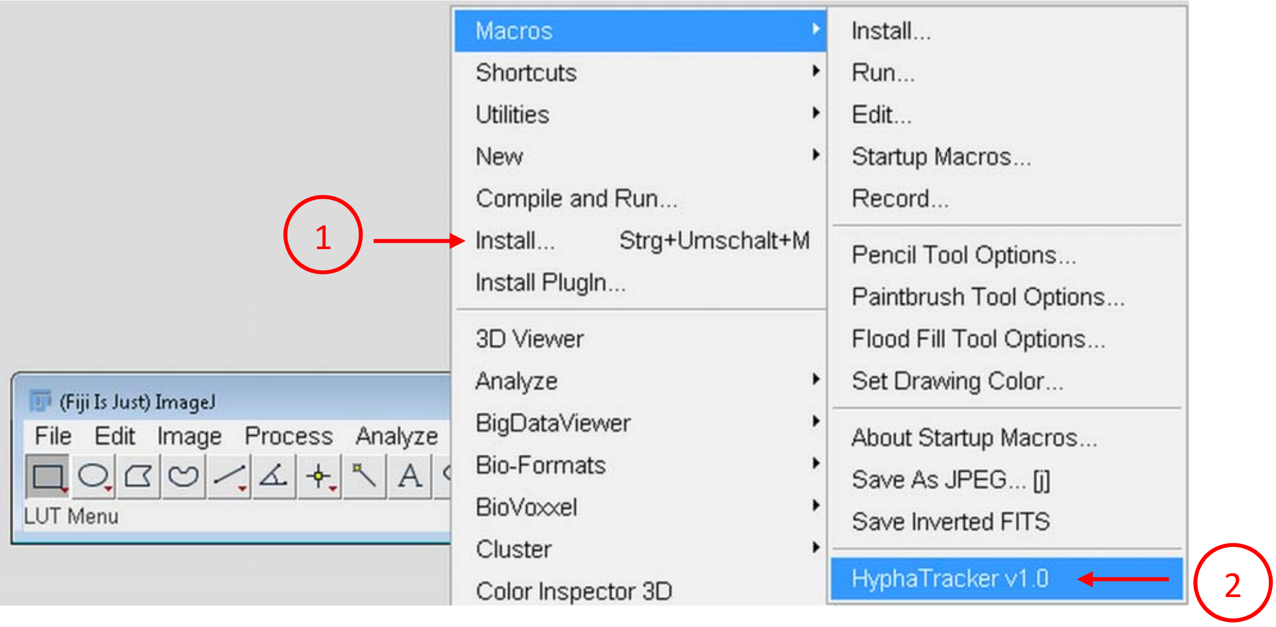

Figure 1. Installation of HyphaTracker.
