# Supplementary material for: HyphaTracker: An ImageJ toolbox for time-resolved analysis of spore germination in filamentous fungi
Source: Sci Rep. 2018 Jan 12;8:605. doi: 10.1038/s41598-017-19103-1 (PMC5766585; doi:10.1038/s41598-017-19103-1)
Supplement: Supplementary file 3 — Supplementary Information [file 41598_2017_19103_MOESM3_ESM.pdf]

Supplementary Information -

HyphaTracker: An ImageJ toolbox for the time-resolved analysis of spore germination in filamentous fungi

Michael Brunk, Sebastian Sputh, Sören Doose, Sebastian van de Linde, Ulrich Terpitz

1 Detailed explanation of the HyphaTracker graphical user interphase (GUI)

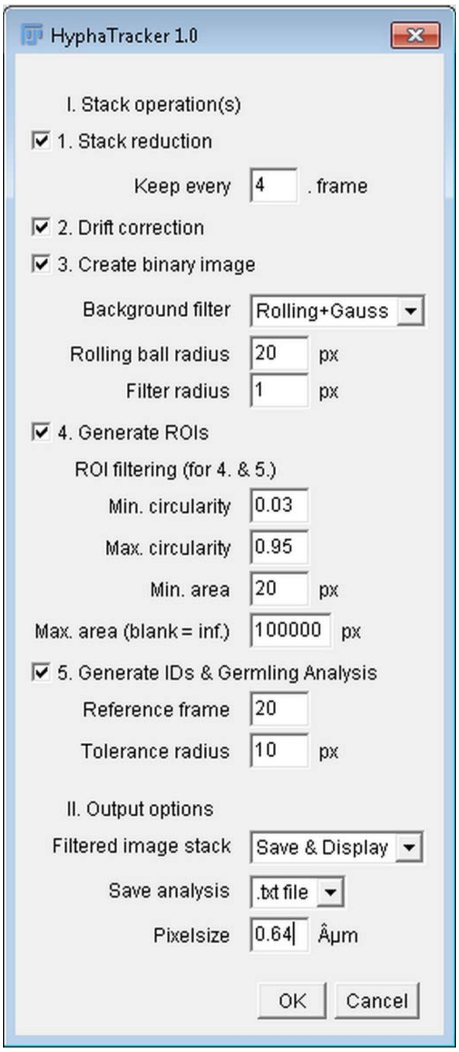

Fig. S1 Graphical user interphase of HyphaTracker in Fiji[2]. For details see text. The Fiji logo is licenced under the Creative Commons Attribution-ShareAlike 3.0 License (https://creativecommons.org/licenses/by-sa/3.0/).

Once HyphaTracker is started a graphical user interphase is shown, which allows the user to select each of five options for image stack analysis. Every operation is a standalone feature and thus can be used independently from the other options, or the elected options can be performed sequentially.

1. **Stack reduction:** The number of frames within the current image series analysed during the HyphaTracker analysis can be reduced by entering the factor by which the entire stack is reduced (e.g. 4 means every 4th frame is kept).

2. **Drift correction**: Allows for correction of x-y-drift using immobilized objects as a reference (e.g. impurities like dust in the image).

3. **Create binary image:** The background is subtracted and the image is transformed from 16 bit depth to binary using customized settings.

4. The **Generate ROIs** function uses the ImageJ[1] Particle-Analyser to generate region of interests (ROIs) that can be filtered according to the customized parameters.

5. While the last-mentioned steps produce an image series suitable for the germination analysis, the **Generate IDs & Germling Analysis** feature contains the sorting core algorithm where reference frame and the radius can be defined.

In general, the output option can be chosen to be saved as text file and in addition also as filtered image series. Furthermore, the pixel size can be defined to reveal data in micrometre dimension. The following paragraphs will guide the reader step by step through the features of the HyphaTracker macro.

1.1 Stack reduction

When recording the germination dynamics for visualisation in movies, high amount of data may be generated resulting in image series of several hundred images of about 1GB size in 16-bit resolution. However, for data analysis often much fewer amount of data are required (See also

Supplemental Fig. S7). Thus, it might be advantageous to reduce the number of frames to be computed before starting the analysis. This data reduction will greatly speed up the analysis by HyphaTracker because less frames must be compared. For example, in our experiment with *F. fujikuroi* conidia a frame rate of 12 frames h^{-1} was chosen resulting in image series of 184 images. In our data analysis we used only every 4th image, reducing the images to be analysed to 46.

1.2 Drift correction

This feature allows the user to correct for x-y stage drift which might occur during the acquisition. This drift correction should be frequently used, as during the germling analysis process ROIs in the first and last frame must correspond to each other. Thus, the lateral position of the conidium is essential for the determination of the germling ID and exclusion of crossing events. To perform the drift correction, an immobile reference point is required. The drift correction is based on monitoring the trajectory of an immobile particle. After selection of a ROI, the image is inverted and smoothed with a Gaussian filter followed by identifying the centre of the particle using ImageJ's "Find Maxima" function. This is done for every frame thus generating a trajectory, which is also displayed. Once accepted by the user, the trajectory is used to translate the full TIF-stack image wise. We used non-germinating spores or cell debris, immobilized on the glass surface as a steady reference for the drift correction. Such reference particles could also artificially be added to the conidia suspension to ease the drift correction. The usefulness of the particle can be justified by the user by the trajectory of the x,y-coordinates that is shown before calculation. After calculation, cropping the stack is recommended as the translation generates black edges. The cropped section from the corrected stack will be used for further analysis. Stack reduction and Drift correction can easily be performed in one step and it is recommended to save the adapted image series before further processing.

1.3 Binary image generation

To ensure good contrast and resolution, by default image stacks would be recorded in 16-bit resolution (as we did in our experiments). This routine now generates a binary image stack from the 16-bit data to make it suitable for further germling analysis.

First the background is filtered to reduce the noise within the picture without losing information on the conidia or their germlings. The pre-settings encompass a combination of rolling ball (standard radius 20px) and Gaussian filter (standard radius 1 px). This is the preferred filter combination but further options are selectable like both aforementioned filters alone and median or mean.

Then the binary image is generated by using the standard ImageJ threshold function (Image>Adjust>Threshold…). The threshold level can either be determined automatically or adapted manually to separate the germling from the background. Though at that point a useful setting is suggested by the ImageJ program, one may manually adapt the threshold-routine to gain better results. Once accepted, the thresholded image is then converted into a binary image.

We used the current ImageJ default thresholding function, an iterative procedure based on the isodata algorithm[3], which was sufficient for our acquisitions. This auto-threshold is done every time, but the user can select alternative methods such the one of Huang, which uses Shannon's entropy function. It should be emphasized that correct adjustment of the threshold is critical, as low threshold may result in high noise, while high threshold may lead to holes in the depicted conidia / hypha areas. Therefore, it is important that the user tests different methods to obtain optimal segmentation.

Remaining noise in the binary image can be removed via the binary filter "Despeckle" in ImageJ. More details regarding this process can be obtained from section 3 ("Choosing the right settings"). If every parameter is chosen in a proper way, the conidia / hyphae will be shown in contrast to the background in the newly generated binary image.

1.4 ROI generation

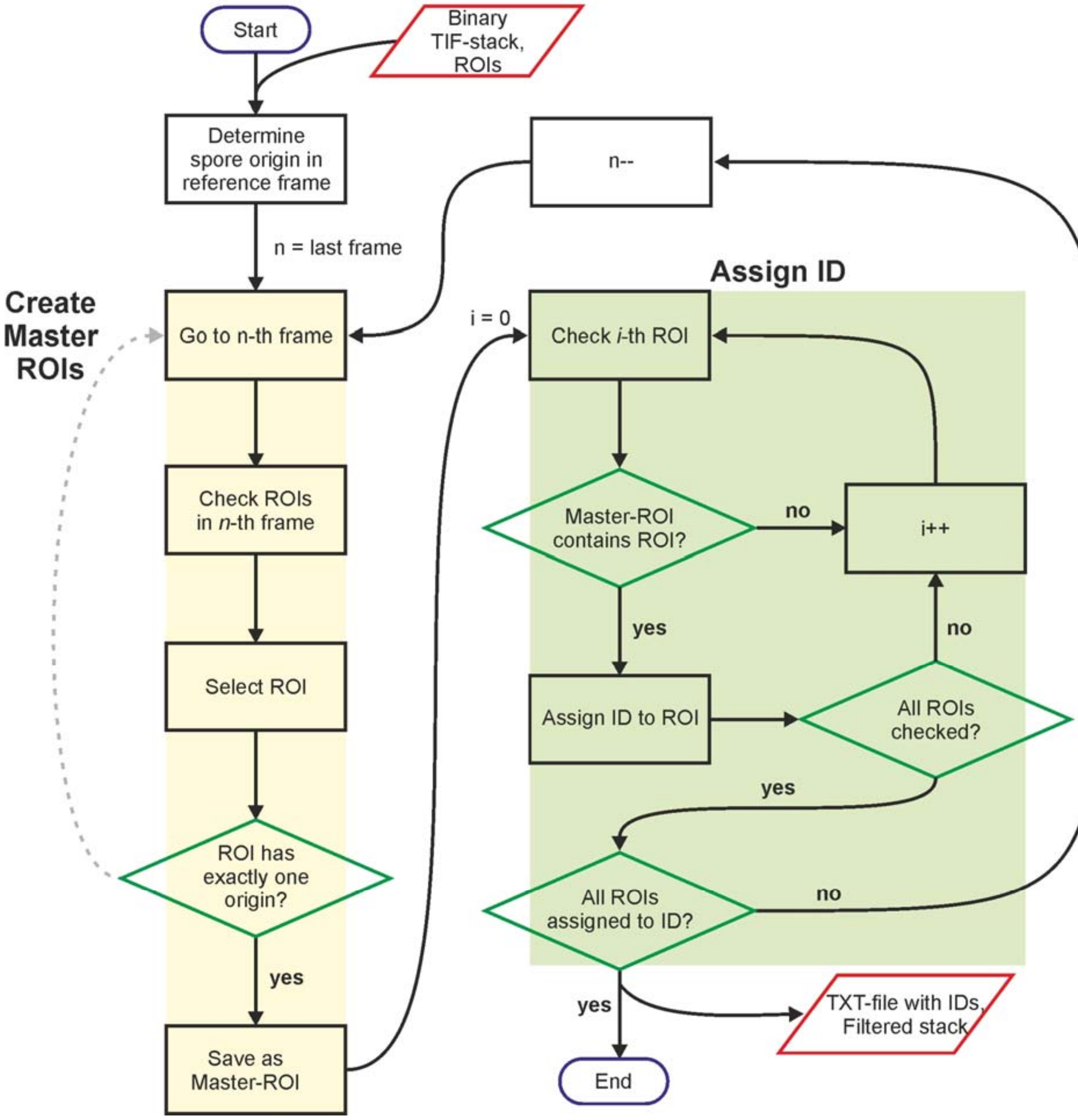

Fig. S2 Flow chart of the ID generation and germling analysis feature of HyphaTracker. The ROIs that were detected in a binary image time series are analysed during this process. *Master-ROI creation:* First a reference frame is determined for the occurrence of non-crossing single spores, then in the last frame, ROIs are analysed for their number of origins. If a ROI present in the current last frame has only one origin, it is saved as Master-ROI. If more than one origin can be assigned, the next earlier frame will be analysed. The procedure is continued until the respective ROI can be assigned to only one single origin. *ID-Assignment:* Any ROI of every frame is analysed if it is matching with a previous Master-ROI. If so, the current ROI is assigned to the corresponding Master-ROI, if not, a previous frame is analysed. If all frames are analysed in this way, new master ROIs are generated and compared to the remaining ROIs until the whole stack was analysed. The text file with IDs is given and the filtered stack is presented/saved.

The ROIs are generated by the tool "Particle Analyzer" implemented in ImageJ. By this feature every conidium/germling is detected as one associated pixel group. Once ROIs are generated, HyphaTracker allows filtering the ROIs for their circularity as well as for their minimal and maximal area. These features are important to avoid false-identification of ROIs that do not represent spores or germlings (remaining noise signal), and to reduce the amount of data to be analysed subsequently.

Circularity of 1 represents a spherical / round shape, whereas 0 represents a completely polarized shape. Many fungi, like *F. fujikuroi* exhibit conidia with strong polarity thus circular structures are unlikely to present conidia and thus can be neglected prior to the analysis. In addition, the conidia are expected to exhibit a least size, which can be freely defined in HyphaTracker. In our experiments this value was set to 20 px (see Fig. S1). This was in accordance with the minimal value of non-germinated spores of *F. fujikuroi* (7.67 μm^2; n=104).

1.5 ID generation & Germling analysis

The "Particle Analyzer" outputs a list of ROIs specified by defined parameters, but for a dynamic analysis it is necessary to assign the ROIs to certain conidia / germlings. The core of HyphaTracker analyses and sorts ROIs in three consecutive steps consisting of the

i) Determination of ROI origins,

ii) Determination of master-ROIs, and

iii) Assigning IDs to ROIs.

For the determination of ROI origins, a reference frame is defined in which all non-germinated spores are visible (dialog: *Reference frame*). This frame must be defined by the user. From this frame ROI coordinates are extracted that serve as origins. The reference frame, which refers to the unprocessed stack is automatically recalculated if data reduction is applied.

In a next step master-ROIs are determined. A master-ROI is a ROI of a hypha with maximum area and only a single spore origin; it is searched backwards from the end of the image stack, where ROIs are discarded that have more than one origin. Consequently, eventually crossing hyphae are neglected at the point when they contact each other and thereby false determination of the hyphal area is avoided. Finally, all ROIs are assigned to a master-ROI, resulting in different integers for each master-ROI (ID). In this procedure it is critical, that the drift has been corrected in advance.

The resulting data are not only displayed in a result table listing ROI, area, frame, position, and germlingID but optionally also graphically represented in a newly generated, filtered binary image time series, allowing for the analysis of the germination dynamics (dialog: *Filtered image stack*).

2 The Usage of HyphaTracker – an example

We use the file HT-teststack-01.tif, which is provided at https://go.uniwue.de/hyphatracker, to visualise the function of HyphaTracker toolbox. The user can re-enact every step by loading this sample stack and following the instructions.

Stack reduction

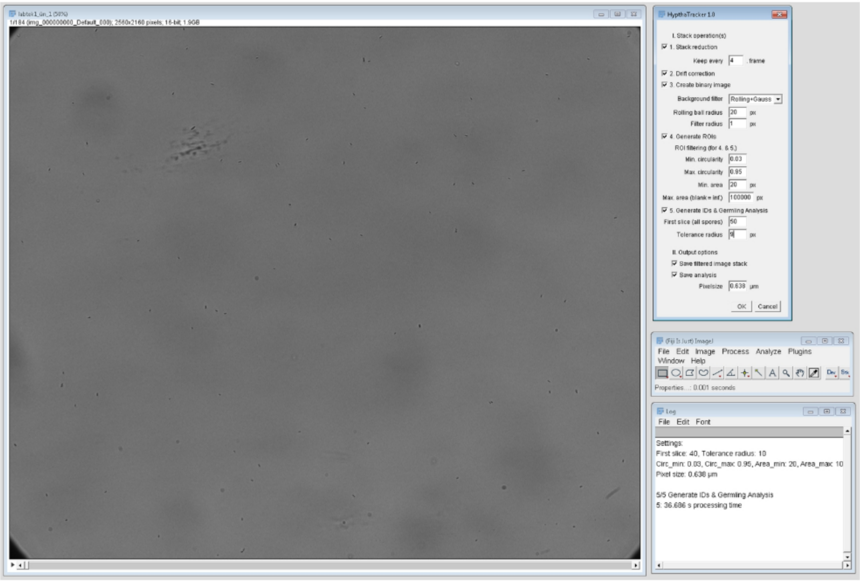

Drift correction

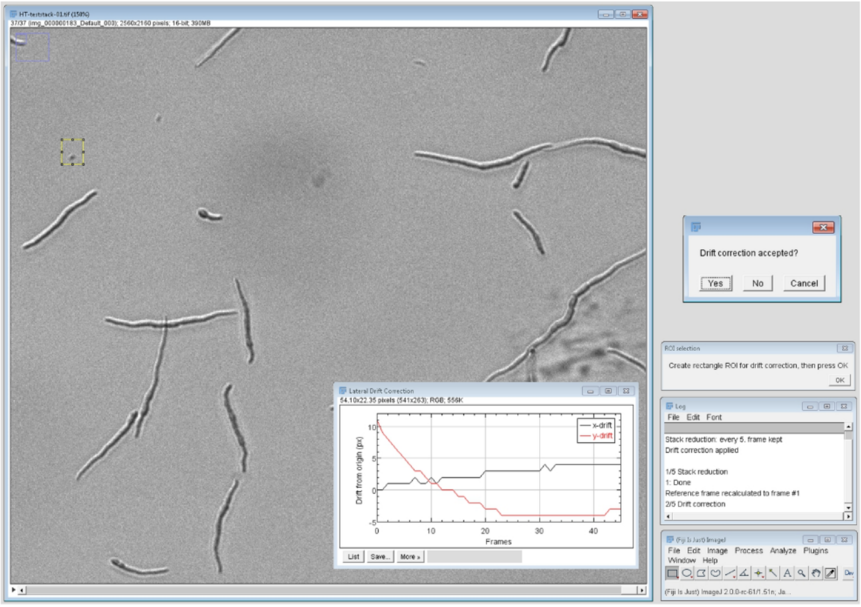

Crop

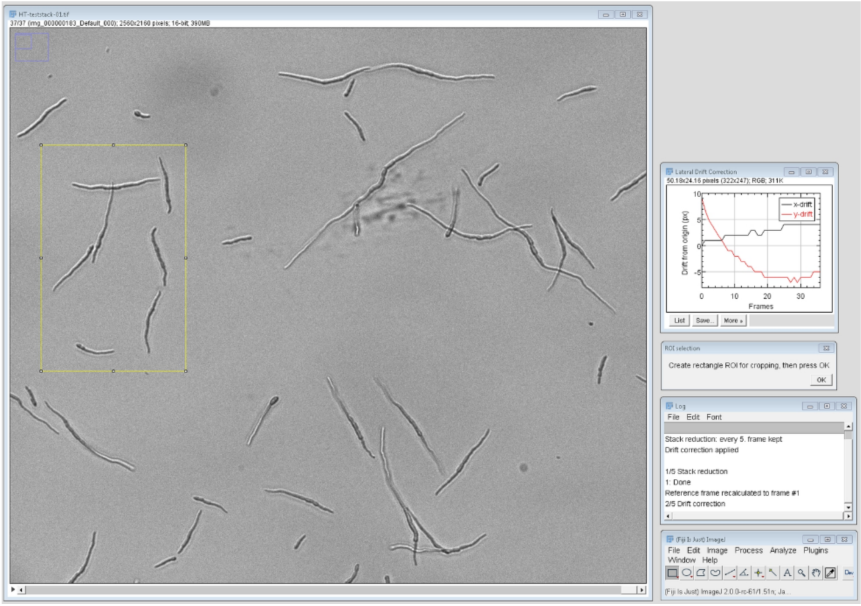

Fig. S3. Stack reduction and Drift correction exemplified with HT-teststack-01. HyphaTracker was used in Fiji[2].

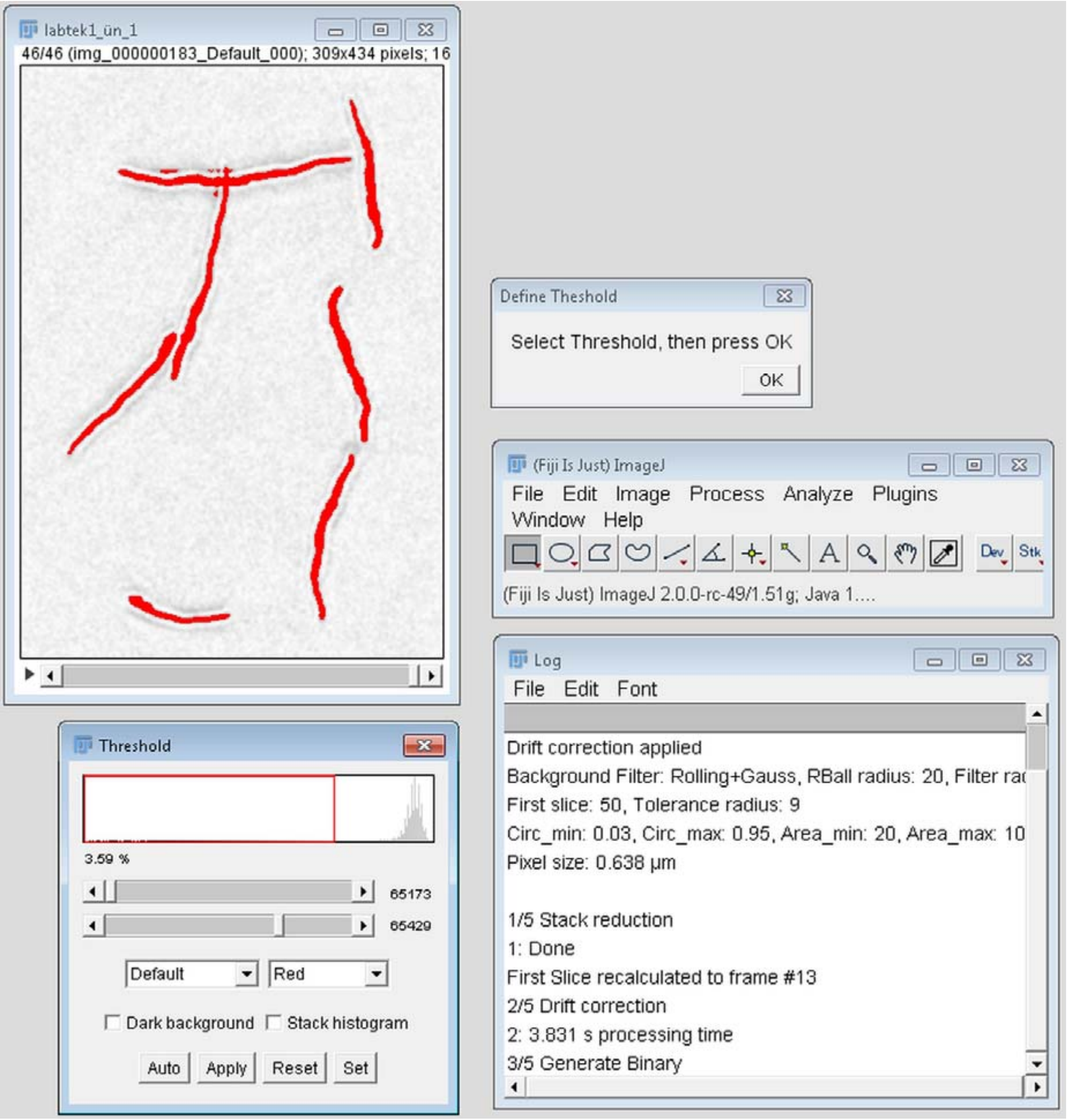

Fig. S4. Threshold adaption while running the "*Create binary image*" option. HyphaTracker was used in Fiji[2].

In the beginning the stack is loaded, HyphaTracker is started, and the parameters are adjusted (Fig. S3). First, the stack is reduced to an appropriate number of data (in this example every 4th frame is kept).

Subsequently the drift correction is started. For this procedure a particle is selected by the user that should be present in each frame and immobilized in the glass surface (see Fig. S3 middle). Before drift calculation starts, a trajectory of the x-/y-coordinates is shown. By that the user can justify the usefulness of his selection and will be asked to approve the start of calculation. Once drift calculation is finished, HyphaTracker will prompt the user to create a rectangular selection for cropping to remove the edges of the image series.

In our example we cropped only a small section containing a few hyphae to better understand and analyse settings in the later steps (see also Fig. S4, and section 3).

If in the beginning all steps were activated, HyphaTracker will now automatically start with the background reduction and binarisation routine. In the next step one will have the possibility to

adapt the threshold accordingly, though in most cases the default parameters are fine (see also section 3.3).

Once the binary image is constructed in the subsequent step 4 of HyphaTracker the ROIs are detected and filtered according the parameters that can be chosen in the beginning (Fig. S5). In our example we chose a circularity of min. 0.03 and max. 0.95 with a minimal area of 20 pxand maximal 100,000 px². In general, this step is very fast. Thus, it is advised to save the binary image series and to run step 4 and 5 conjointly in HyphaTracker.

Step 5 contains the filter-routine and correct settings are required to get convenient results. For a more detailed analysis how parameters are affecting the outcome of the analysis please consult section 3.5 and Figure S11, which is directly related to this example.

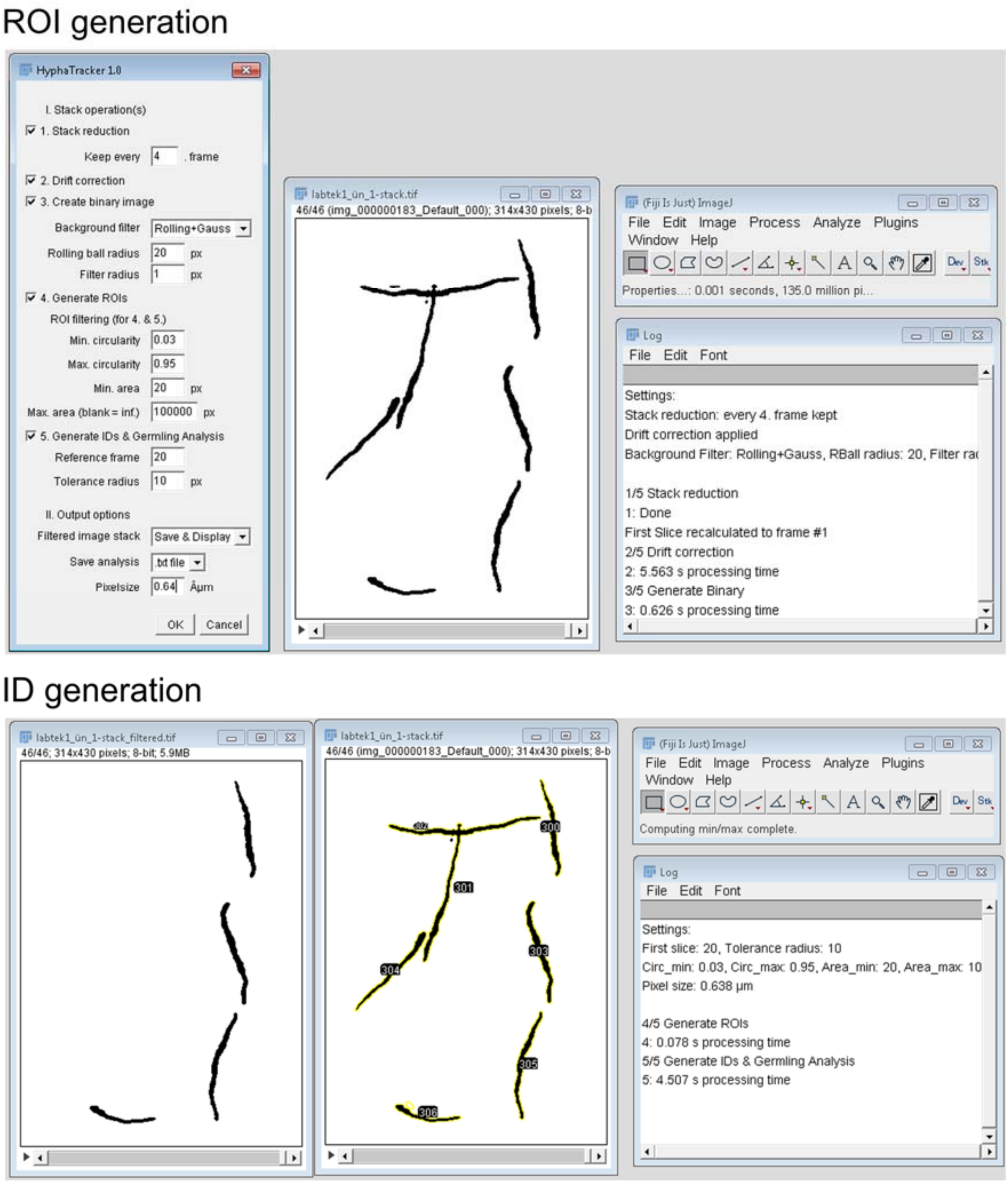

Fig. S5. ROI generation and Germling analysis. HyphaTracker was used in Fiji[2].

3 Choosing the right settings – how the parameters influence the analysis

The consequence of parameter setting is exemplified in the following section using the image stack HT-teststack-02.tif, which is provided at https://go.uniwue.de/hyphatracker to allow the reader to follow these instructions. In some exceptional cases further examples are chosen to explain certain settings in detail.

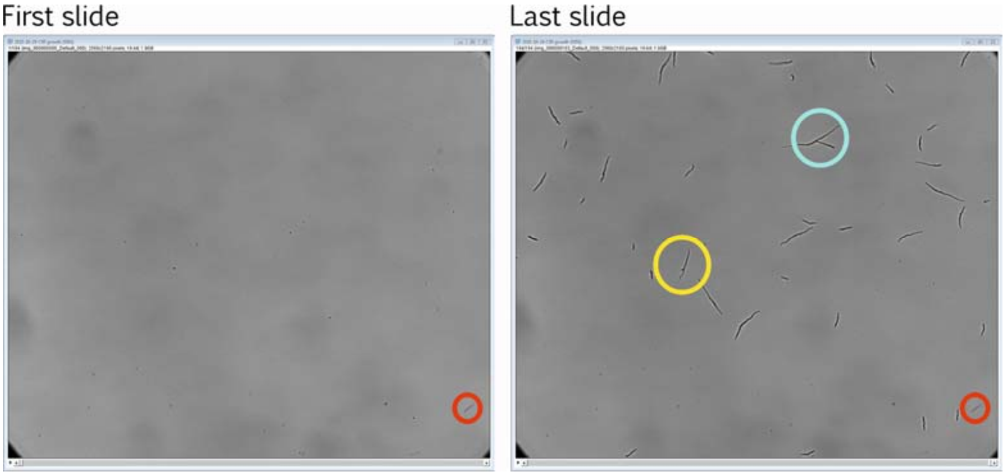

Fig. S6. Image stack "HT-teststack02.tif". The first and last slide is shown. Circles highlight a scratch in the microscopic lens (red), a branching hypha (yellow) and crossing (cyan) germlings. HyphaTracker was used in Fiji[2].

- Load the 16-bit image series HT-teststack02.tif. [File → Open …] Note: If you import image sequences instead, it is essential to use non-virtual stacks for the analysis since virtual stacks do not apply changes done with the HyphaTracker Macro.
- The resulting image series has a size of 1.9 GB and should contain a series of 184 images with a size of 2560 x 2160 px and a depth of 16-bit.
- Inspect the stack for growing hyphae. In Figure S6 you can clearly note the blurry spots in the background, a scratch on the microscopic lens (lower right: red cycle) as well as non-/ growing, crossing (cyan cycle) and branching hyphae (yellow cycle).
- Run HyphaTracker in ImageJ. For detailed instructions please consult the Installation Guide included in the macro-package. If no image series is open, HyphaTracker will ask for opening a suitable image stack.
- A GUI will appear (Fig. S1). The user can now apply several options (Compare with standard settings in the SI). Except for the options *Stack reduction* and *Drift correction* as well as the options *Generate ROIs* and *Generate IDs & Germling Analysis*, for the sake of flexibility, we do not recommend combining multiple options at a time.
- As a first step the number of images in the opened image series might be reduced and the series might be corrected for drift (Compare with Details in SI). In this example, the stack will be reduced to every 4th image.
- After each successful operation the user will be informed about the current progress within a log-output.

Note: We recommend saving the processed image series after each step to allow reprocessing if required. For option 5 (*Generate IDs & Germling Analysis*) it is advised to enter the Scaling factor for the pixel size ratio into the macro. In our case the ratio was about 0.64 µm/px.

3.1 Stack Reduction

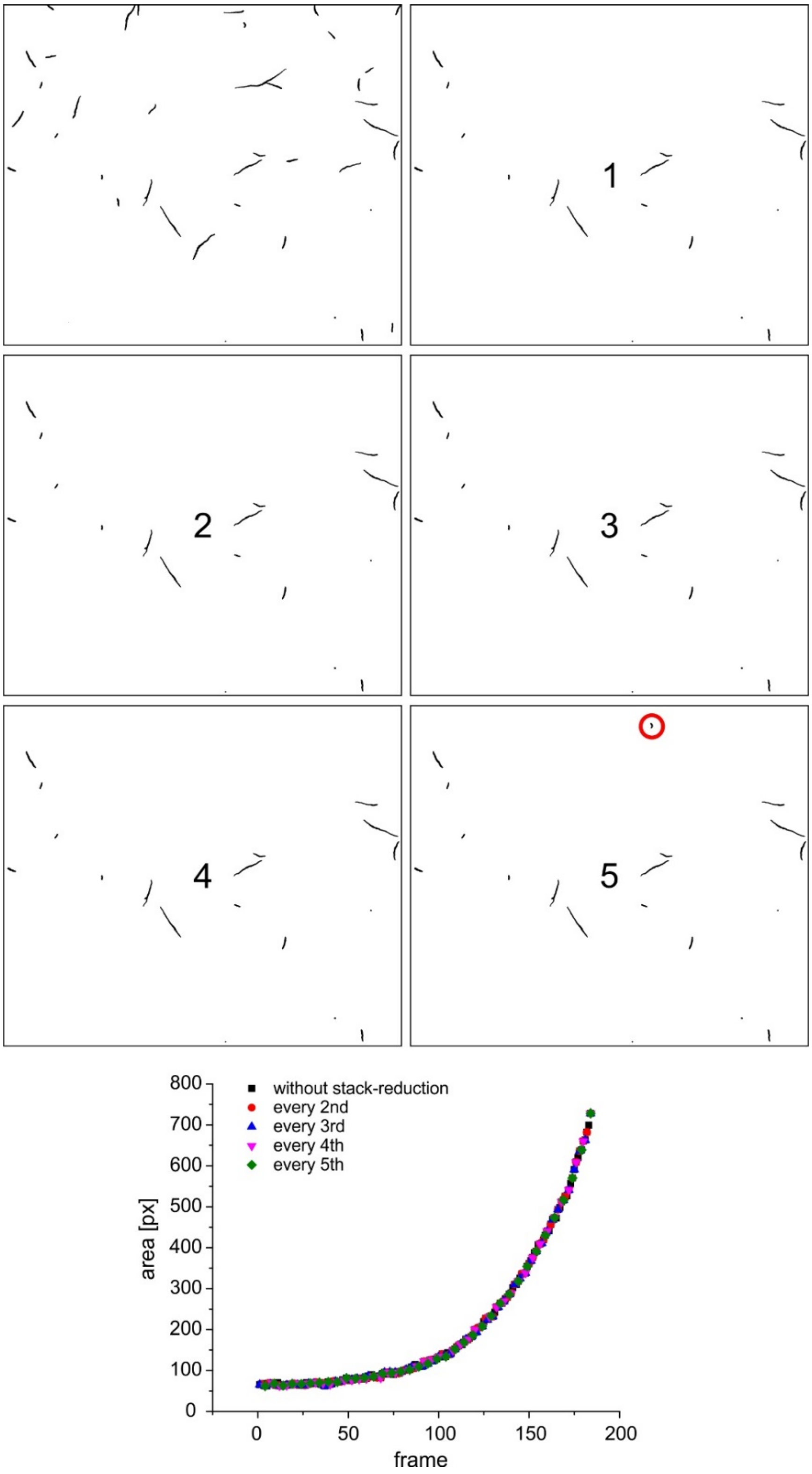

Fig. S7 Consequence of the stack reduction on the filtering routine of HyphaTracker. The last image of the image series HT-teststack2 before filtering (top left) and after filtering as indicated is shown. The analysis was performed with a tolerance of 8 px using every (1), every second (2), every third (3), every fourth (4), or every fifth (5) frame. The reference frame was set to 40, 20,13,10, or 8, respectively. In case of the analysis of only every 5th frame an additional hypha is observed (red circle). Bottom: The representative areal growth of one germling (ID4) is shown in dependence of different reductions as indicated.

Stack reduction might be required to speed up the image processing. It is important to adapt the reference frame used in the germling identification accordingly. In general, we found that the

stack reduction does not influence the filtering routine of HyphaTracker. While analyzing HT-teststack2 with different reduction settings, we found only one discrepancy in the scenario in which every 5th frame was used, here one additional hypha was detected which was due to a shifted reference frame.

3.2 Drift correction

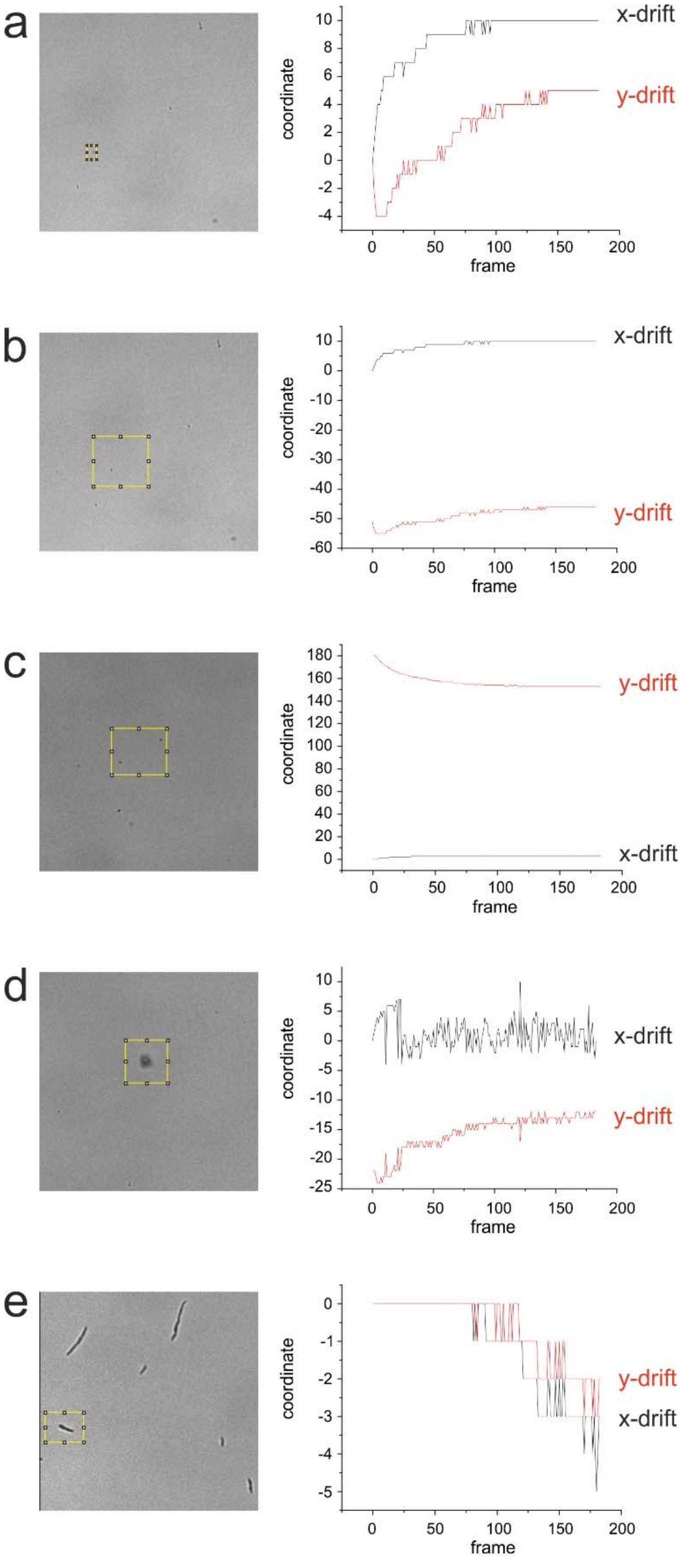

Fig. S8. Drift correction. Influence of the selected reference on the success of drift correction. a. A single particle is chosen for the correction. The rectangular ROI is created as small as possible to involve the particle in all frames. The trajectory shows an optimal drift correction behavior with towing saturation curves. b. A ROI was created encompassing much background compared to the particle. Though the curves show the same shape, the y-coordinate is shifted about 50px to the negative, leading to loss of the lower border during correction. c. Two reference objects were chosen instead of one single element leading to a strong loss of >150 px in the y-coordinates during drift correction, d. Big, fluffy structures are not suitable for drift correction, the trajectory shows high noise, which will negatively affect the analysis by hyphatracker. e. Living conidia or hypha are not suitable for correction.

3.2.1 Influence of the selected reference in the accuracy of drift correction.

The reference for drift correction needs to be carefully selected to avoid erroneous data analysis. Especially one must avoid choosing artefacts which are part of the microscope optics and do not move throughout the entire stack series e.g. steady particles & scratches because suchlike selection would not at all influence the drift correction.

The characteristics of the reference object might influence the drift correction. This can easily be monitored by the x,y-trajectory that is displayed during the drift correction routing. The best results are obtained when small particles like non-germinating spores are chosen (Fig. S8a), in this case the correction will be relatively precise, also due to the limited changes in the shape during a potential z-drift (HT-teststack-04.tif; https://go.uniwue.de/hyphatracker). The size of selection around a suitable particle might affect the selection routine leading to strong displacement of the complete image series as visualized by expanded black space before cropping routine and thus to reduced amount of accessible data (Fig. S8b). One might think that selection of multiple reference objects might improve drift correction, but this is not the case as exemplified in Figure S8c (HT-teststack-03.tif; https://go.uniwue.de/hyphatracker). Indeed, multiple reference selection will lead to either no correction or strong shift of the entire series. In the example Fig. S8d a big particle is chosen e.g. crystal. Suchlike selection will result in very unprecise correction, where the stack gets shaky, probably due to the out-of-focus changing size of bigger particles. It is not recommended to use growing hyphae as reference (Fig. S8f) since orientation and speed of growth will affect the drift correction.

3.2.2 Consequence of skipping correction step

Depending on the hardware and data acquisition especially in the first two hours the sample and/or the microscope stage often underlie drift. In case of drift data interpretation by HyphaTracker will be strongly influenced by displaced germlings. Therefore, drift correction is highly recommended if the microscopic system is not equipped with a suitable stabilization system.

3.3 Create binary image

For analysis of *Fusarium fujikuroi* strains the background filtering option ‘Rolling+Gaussian’ with a standard setting of 20 px for the Rolling ball radius and 1 px for the filtering radius have been used. Yet, further options are given and should be checked for resulting picture quality if required.

Depending on the image quality, manually adjusted thresholding might be necessary. However, a threshold that was set too high or low might have a huge impact on further analysis, namely under- or overrepresentation of potential hyphae (Fig. S9).

We recommend saving the binary output before proceeding to test for ideal settings in the following options.

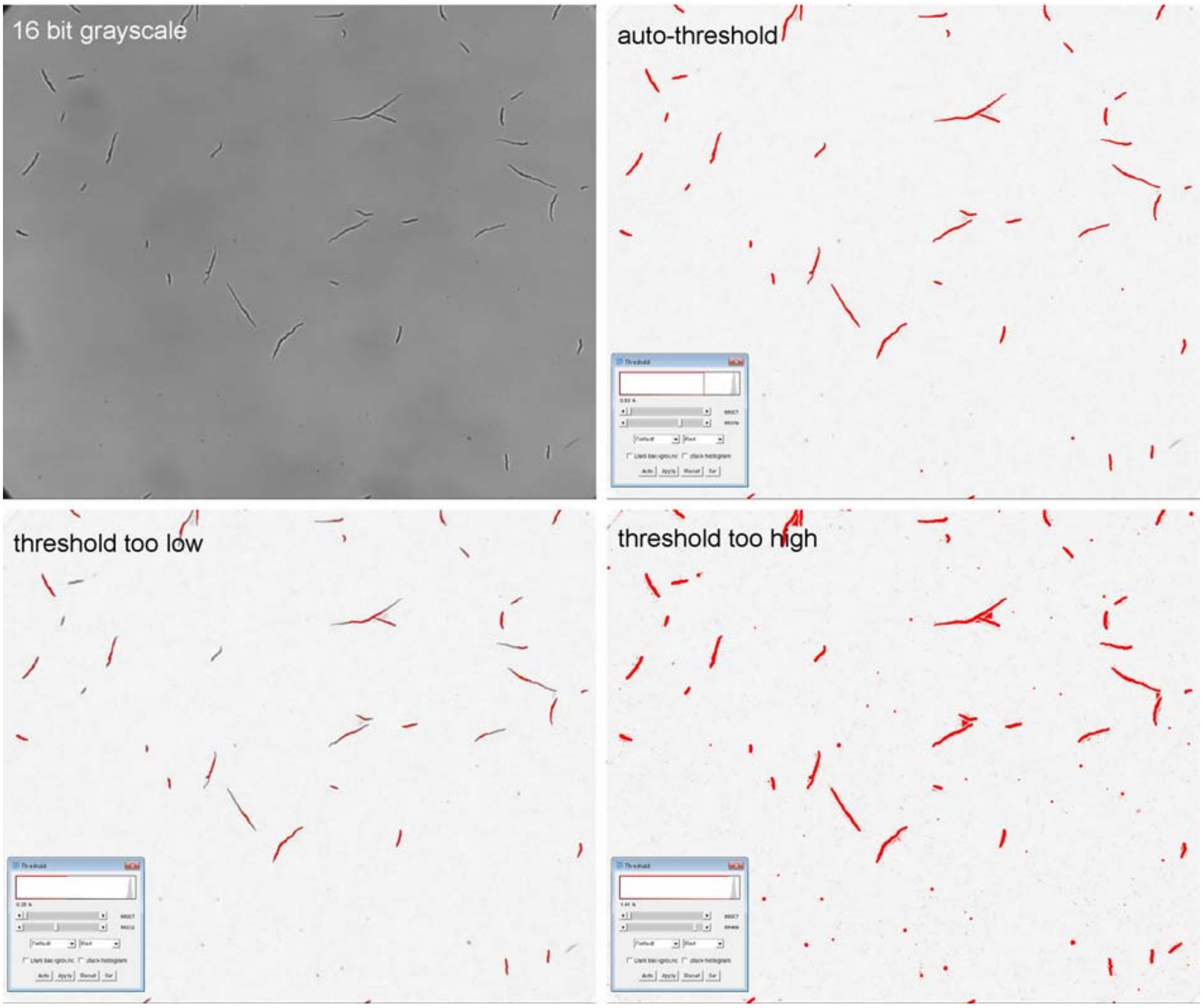

Fig. S9. Importance of the threshold during the *Create binary image* routine. In most cases using the 16-bit grayscale image stack (left, top) the default auto-threshold will end up with a satisfying result by rejecting most of the non-spore particles while fully representing the actual spores (top, right). If the threshold is set too low (bottom, left), the areal space of spores might not properly be filled and less spores and background particles might end up in the analysis. In contrast, when the threshold is set too high (bottom, right), it will result in further shading of non-hyphal areas like shadows, scratches and air bubbles.

3.4 Generate ROIs

The "Particle Analyzer" of ImageJ is used to generate several hundreds to thousands of ROIs that are then used for further analysis in the ID generation. The user should take care with the boundaries settings, which can be defined for the min/max values of area and circularity. In the early development the conidia / germlings might exhibit very small area or almost round shape. If the confinements are set too strict in (undesired) consequence useful hyphae might be excluded from analysis. Also note, that the ROI output is given in px. The option "Pixelsize" only affects the output text file of "*Generate IDs & Germling analysis*".

Based on previous experiences a minimum circularity of 0.03 and a maximum circularity of 0.9-0.99 have been chosen for pre-filtering spores from bubbles and other particles. Also, a minimum area of 20 px and a maximum area of 100,000 px^2 was chosen for pre-filtering, dismissing smaller areas from the binary picture which might be caused by an uneven illumination.

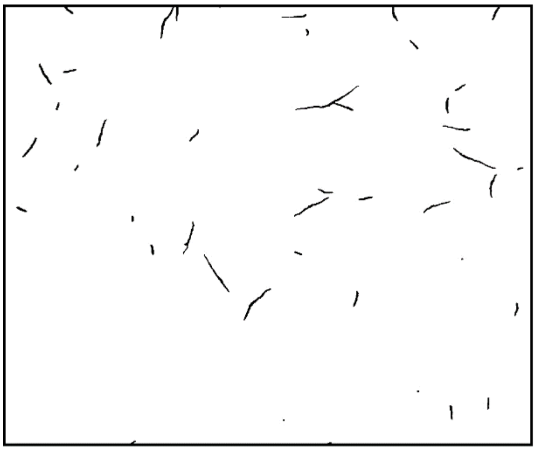

unfiltered binary

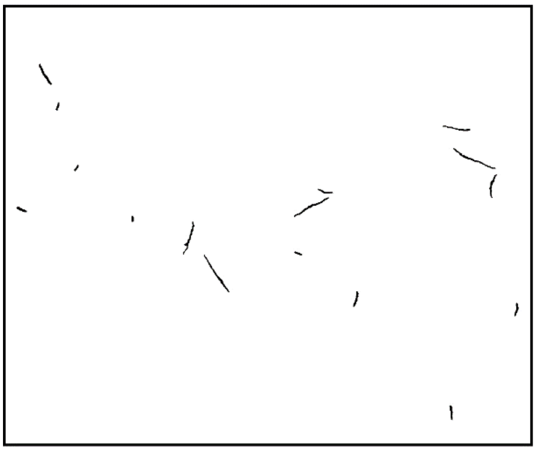

tolerance radius 10 px
reference frame 5

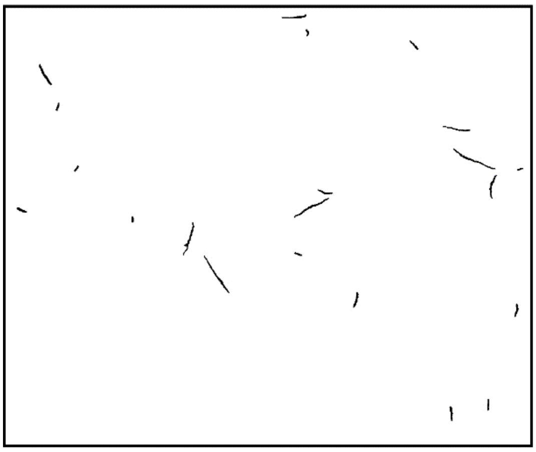

tolerance radius 10 px
reference frame 20

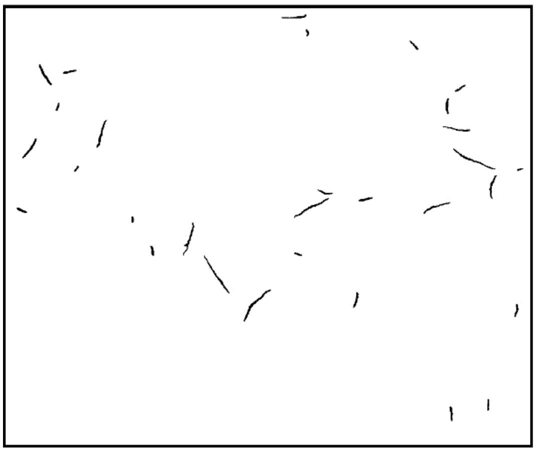

tolerance radius 10 px
reference frame 40

Fig. S10. Comparison of original frame vs filtered output at a sorting radius of 10 px using either reference frame, 5, 20, or 40, as indicated.

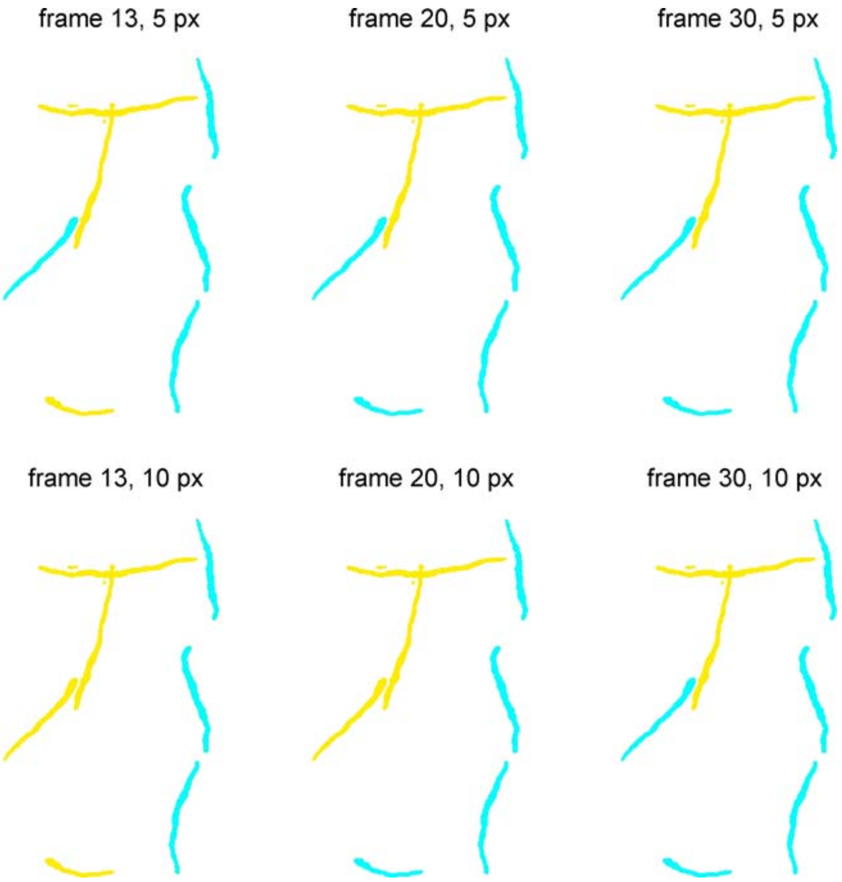

Fig. S11. Removal of crossing hyphae from the data analysis. The filter routine is influenced by the choice of the reference frame and the tolerance. The section shown in Fig. S4 was analysed using different settings as indicated. Cyan: Hyphae present after filter routine. Yellow: Hyphae rejected by the filter routine.

3.5 Generate IDs & Germling analysis

This option requires the output from the previous option (3.4.). The mechanism of ID generation and germling analysis is explained in detail in section 2. The chosen reference frame and the size of tolerance radius strongly influence the outcome of this routine as shown in the examples in Figs. S10 and S11.

In general, for successful ID sorting a reference frame of 20 (after reduction by a factor of 4 from Original series) and a radius of 10 px have been chosen. It is important that the reference frame does not contain any crossing spores / hypha.

If settings are chosen in a right way, within the resulting image crossing hyphae should be dismissed while uncrossed hyphae are still present. The later the chosen reference frame the more IDs will be taken for the final output. This is especially the case for analyses in which conidia appear later in the image series or hyphae grow out of the focal plane

A general challenge is the phenomenon that in the bright-field microscopy often the middle of a hyphae appears paler than its edges, leading to their appearance as holed structures in the binary image. Therefore, the ImageJ binary filter “Fill holes” was implemented into the HyphaTracker code as part of the “detect germling” routine recovering holes in the hyphae. This might also lead to false filling in crossing area or between hyphae and particles. Therefore, it is suggested to manually review the output data. It should be noted that the object labelling itself does not take into account image artefacts. Consequently, confinements for germling data used in the meta-analysis should be strict.

In this respect, it is important to verify that only hyphae are used for further analysis that are present over the complete germination period. If a high amount of conidia is used during germination, more crossing events are expected and thus a selection occurs towards the shorter, not crossing hyphae. For that reason, it is critical to balance the number of conidia to be analysed between as many germling as possible and as few crossing events as possible.

4 Supporting Figures of experimental data

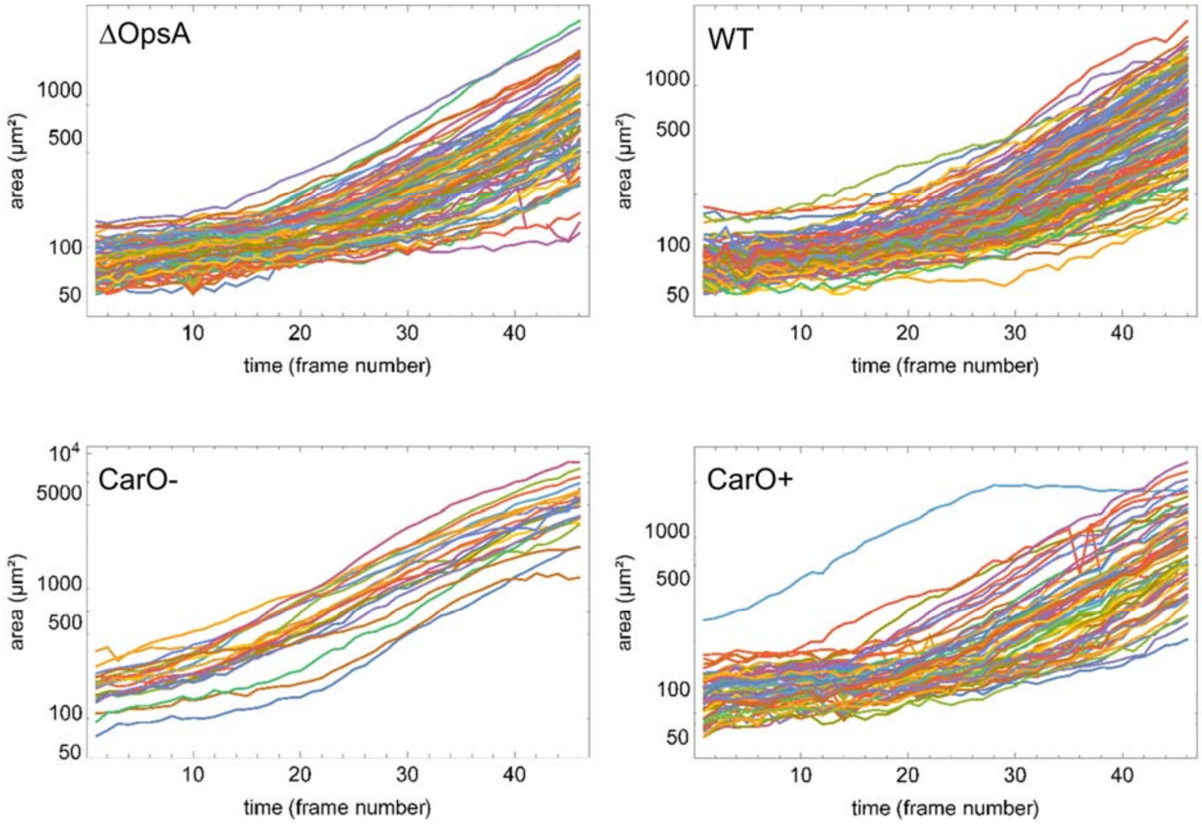

Fig. S12. Hyphae areas generated with HyphaTracker plotted semi-logarithmically as function of time for every single conidia and for each strain as indicated. Note, that the y-scale for the CarO- strain differs from the others. Obviously, germination of the CarO-deficient strain starts earlier and growth occurs faster than in the CarO+ strain, wild type, and the OpsA deletion mutant

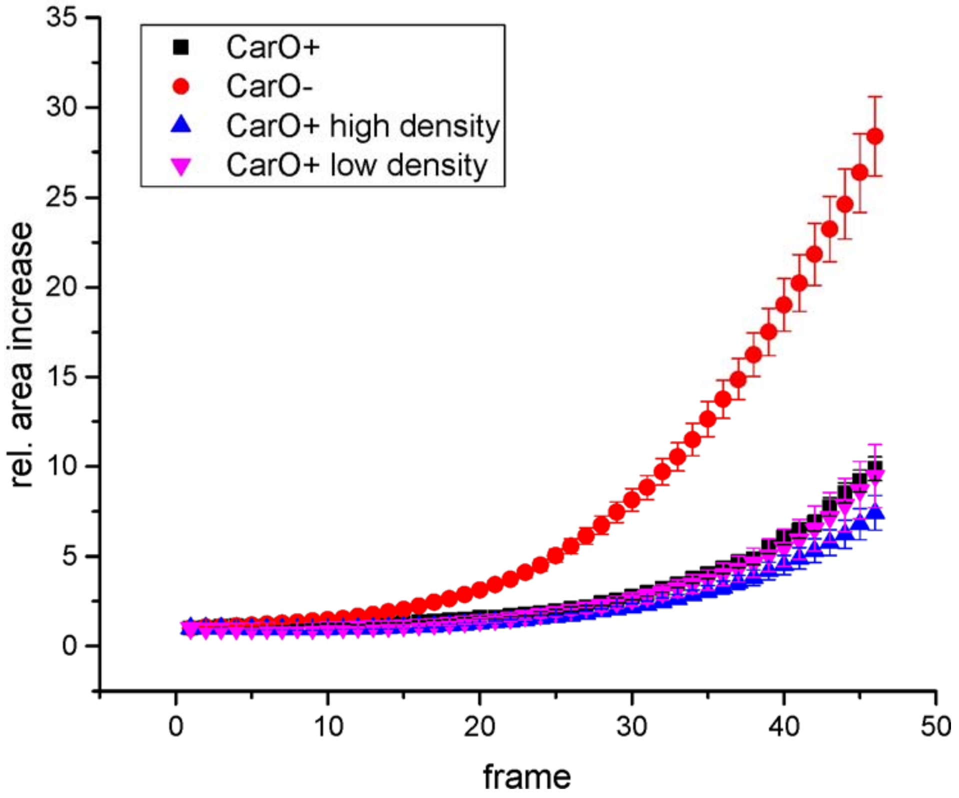

Fig. S13. Influence of conidia density on the conidia germination / germination analysis. For details see main manuscript.

5 Additional supplementary files

Supplementary Software: Program source and Installation guide: “HyphaTracker_v1.0.zip” containing “HyphaTracker_InstallationGuide.pdf” and “HyphaTracker_v1.0.ijm”.

Supplementary data: Sample stacks for practicing: “HT-smallteststack-01.tif” is provided as supplemental file. In addition “HT-teststacks.zip containing ”HT-teststack-01.tif”, “HT-teststack-02.tif”", “HT-teststack-03.tif”, “HT-teststack-04.tif” and “Simulation.zip” containing “testImage_series1_1_1.tif” (SNR 13.3), “testImage_series1_2_1.tif” (SNR 6.7), and “testImage_series1_3_1.tif (SNR 3.3)” can be downloaded from https://go.uniwue.de/hyphatracker.

6 References

1. Schneider, C. A., Rasband, W. S. & Eliceiri, K. W. NIH Image to ImageJ: 25 years of image analysis. *Nat. Methods* **9,** 671–5 (2012).
2. Schindelin, J. *et al.* Fiji: an open-source platform for biological-image analysis. *Nat. Methods* **9,** 676–682 (2012).
3. Ridler, T. W. & Calvard, S. Picture Thresholding Using an Iterative Selection Method. *IEEE Trans. Syst. Man. Cybern.* **8,** 630–632 (1978).

The Fiji logo is licenced under the Creative Commons Attribution-ShareAlike 3.0 License (https://creativecommons.org/licenses/by-sa/3.0/)
